# Notch-Signaling Deregulation Induces Myeloid-Derived Suppressor Cells in T-Cell Acute Lymphoblastic Leukemia

**DOI:** 10.3389/fimmu.2022.809261

**Published:** 2022-04-04

**Authors:** Paola Grazioli, Andrea Orlando, Nike Giordano, Claudia Noce, Giovanna Peruzzi, Behnaz Abdollahzadeh, Isabella Screpanti, Antonio Francesco Campese

**Affiliations:** ^1^ Department of Experimental Medicine, Sapienza University, Rome, Italy; ^2^ Department of Molecular Medicine, Sapienza University, Rome, Italy; ^3^ Center for Life Nano- and Neuro-Science, Fondazione Istituto Italiano di Tecnologia (IIT), Rome, Italy

**Keywords:** MDSC, T-ALL, Notch, IL-6, tumor microenvironment

## Abstract

Notch receptors deeply influence T-cell development and differentiation, and their dysregulation represents a frequent causative event in “T-cell acute lymphoblastic leukemia” (T-ALL). “Myeloid-derived suppressor cells” (MDSCs) inhibit host immune responses in the tumor environment, favoring cancer progression, as reported in solid and hematologic tumors, with the notable exception of T-ALL. Here, we prove that Notch-signaling deregulation in immature T cells promotes CD11b^+^Gr-1^+^ MDSCs in the Notch3-transgenic murine model of T-ALL. Indeed, aberrant T cells from these mice can induce MDSCs *in vitro*, as well as in immunodeficient hosts. Conversely, anti-Gr1-mediated depletion of MDSCs in T-ALL-bearing mice reduces proliferation and expansion of malignant T cells. Interestingly, the coculture with Notch-dependent T-ALL cell lines, sustains the induction of human CD14^+^HLA-DR^low/neg^ MDSCs from healthy-donor PBMCs that are impaired upon exposure to gamma-secretase inhibitors. Notch-independent T-ALL cells do not induce MDSCs, suggesting that Notch-signaling activation is crucial for this process. Finally, in both murine and human models, IL-6 mediates MDSC induction, which is significantly reversed by treatment with neutralizing antibodies. Overall, our results unveil a novel role of Notch-deregulated T cells in modifying the T-ALL environment and represent a strong premise for the clinical assessment of MDSCs in T-ALL patients.

## Introduction

The signaling of Notch receptors is simple and conserved, although many levels of regulation exist and its outcomes are strictly context-dependent (1–3). In mammals, it starts with the interaction between receptors (Notch1 to Notch4) and ligands (Jagged or Delta family) expressed on neighboring cells. Two sequential proteolytic cuts then lead to the release of the functional intracellular domain of Notch (ICN), which translocates into the nucleus to activate the CSL transcription factor. Notch exerts essential roles in T-cell development and differentiation (4–6), and ICN constitutive activation drives the development of “T-cell acute lymphoblastic leukemia” (T-ALL) (7), as demonstrated in murine models (8–10) and reported in patients (11, 12). Many efforts have been produced to dissect the oncogenic role of Notch inside T-ALL cells. However, the effects of T-cell-targeted deregulation of Notch signaling on tumor environment remain underexplored.

“Myeloid-derived suppressor cells” (MDSCs) represent a small and heterogeneous group of immature/progenitor myeloid cells. In mice, they are broadly identified as CD11b^+^Gr-1^+^ cells (13) and are expanded/activated in the tumor environment, where they suppress host immune responses, facilitating cancer progression (14). MDSCs have been described in detail in solid tumors (15, 16). Instead, studies are limited and with conflicting results in hematological malignancies, and nothing has been reported for T-ALL (17, 18), so far.

Notch may regulate MDSC behavior in a different context (19, 20). Notch-signaling inhibition in Gr-1^+^ cells facilitates the aberrant production of MDSCs in tumor-bearing mice and cancer patients (21, 22). In contrast, other groups reported that Notch-signaling inhibition, by using GSI or anti-jagged antibodies, limits the function of MDSCs in cancer settings, thus suggesting a positive role of Notch activation (23, 24). The non-cell autonomous influence of Notch activation on the myeloid compartment was already reported in T-ALL. Indeed, the constitutive expression of ICN1 in murine bone marrow (BM) progenitors, by retroviral vectors, induces the expansion of CD11b^+^Gr-1^+^ cells in non-transduced populations (25, 26). On the other hand, myeloid cells may directly influence the onset and progression of T-ALL (27). Nonetheless, the possible arising of MDSCs in T-ALL, their crosstalk with leukemic T cells, and the underlying mechanism/s remain largely undetermined.

Here, we demonstrate that functional CD11b^+^Gr-1^+^ MDSCs accumulate in a transgenic murine model of Notch3-dependent T-ALL (*N3-tg* mice), bearing an *lck*-driven constitutive expression of ICN3 in T cells (9). The aberrant CD4^+^CD8^+^ [double positive (DP)] T cells from *N3-tg* mice drive the generation of MDSCs in a coculture system with BM progenitors of *wt* mice, as well as in NSG hosts, upon their adoptive transfer. Furthermore, we suggest that induced MDSCs facilitate, in turn, the proliferation and expansion of DP T-ALL cells. We also show that MDSC induction is IL-6-dependent. Finally, we start extending our observations to humans, showing that Notch-deregulated T-ALL cell lines promote *in vitro* the generation of CD14^+^HLA-DR^low/neg^ MDSCs from healthy-donor PBMCs, through a mechanism that depends on both Notch and IL-6.

In summary, our preclinical studies and preliminary observations in humans could pave the way for exploring MDSCs as a potential target of therapy for Notch-dependent T-ALL.

## Materials and Methods

### Flow Cytometry and Cell Sorting

Single-cell suspensions of thymus, spleen, BM, or peripheral blood from *N3-tg* or *wt* mice were resuspended in PBS; 2% FBS and erythrocytes were lysed with ammonium-chloride–potassium lysing buffer, as described (28); then, cells were stained with surface markers for 30 min on ice, using anti-CD4-PerCPCy5.5 (RM4-5), anti-CD8-APC (53-6.7), anti-CD11b-FITC (M1/70), and anti-Gr-1-PE (RB6-8C5) antibodies, all from BD Bioscience (La Jolla, CA, USA). For IL-6 detection, freshly isolated BM or thymus cells were stimulated for 4 h with PMA 50 ng/ml and ionomycin 1 μg/ml (Sigma Aldrich, St Louis, MA, USA), in the presence of brefeldin A (Golgi Plug, BD Bioscience, La Jolla, CA, USA). Samples were stained with the appropriate surface markers, followed by the intracellular staining with anti-IL-6-PE (MP5-20F3) antibody or isotype control, performed by using Cytofix/Cytoperm kit (BD Bioscience, La Jolla, CA, USA), according to manufacturer’s instructions. For arginase-1 intracellular detection, samples were stained with the appropriate surface markers, followed by the intracellular staining with anti-arginase1-APC (A1exF5) antibody or isotype control (both from Invitrogen/eBiosciences, San Diego, CA, USA), performed by using the FoxP3/Transcription Factor Buffer set (Invitrogen/eBiosciences, San Diego, CA, USA), according to manufacturer’s instructions. Samples were run on FacsCalibur, equipped with CellQuestPro software (BD Bioscience, La Jolla, CA, USA).

Human MDSCs were assessed by using antibodies against CD33-FITC (HIM3-4), CD11b-PE (ICRF44), CD14-PerCPCy5.5 (M5E2), HLA-DR-APC (G46-6), and Fixable-Viability Stain 780 to discriminate dead cells, all from BD Bioscience (La Jolla, CA, USA). The samples were run on a BD-LSRFortessa cytometer and analyzed with the FlowJo software (Tree Star). For IL-6 intracellular detection in human T-ALL cell lines, the cells were cultured 5 h in the presence of brefeldin A (Golgi Plug, BD Bioscience, La Jolla, CA, USA). The cells were then stained with 0.25 μg of anti-IL-6-PE (MQ2-13A5) antibody by using Cytofix/Cytoperm kit (BD Bioscience, La Jolla, CA, USA). To discriminate specific staining from artifactual staining, we used ligand blocking control where anti-IL-6 antibodies (0.25 μg) were preblocked for 30 min with an excess of human recombinant IL-6 (1 μg), prior to the addition to sample, according to manufacturer’s instructions.

For cell sorting, cell suspensions of thymus, spleen, or BM from *N3-tg* or *wt* mice were stained with anti-CD4, anti-CD8, anti-CD11b, and anti-Gr-1 antibodies, as above. CD4^+^CD8^+^ (DP) T, non-DP T (that includes CD4^+^CD8^−^, CD4^−^CD8^+^, and CD4^−^CD8^−^ T cells, sorted from pre-enriched pan T-cell samples), CD4^−^CD8^+^ or CD11b^+^Gr1^+^ subsets were isolated (purity level ≥97%) with a FACSAriaIII cell sorter, equipped with the FACSDiva software (BD Bioscience, La Jolla, CA, USA).

### RNA Isolation and RT-qPCR

Total RNA was extracted with TRIzol reagent (Invitrogen/ThermoFisher, Waltham, MA, USA), and reverse-transcription was performed with High-Capacity cDNA Reverse-Transcription Kit (ThermoFisher, Waltham, MA, USA). The mRNA expression levels of murine Arginase-1 and IL-6 (Mm00475988_m1 and Mm00446190_m1 assay, respectively, by ThermoFisher, Waltham, MA, USA) were determined by TaqMan quantitative real-time RT-PCR performed on cDNA, using the StepOn ePlus™ Real-Time PCR System (ThermoFisher, Waltham, MA, USA), following the manufacturer’s instructions. Data were analyzed by the ΔΔCt method; murine HPRT was used as reference.

### ELISA

BM supernatants were obtained by flushing bones in 500 μl of ice-cold PBS; after centrifugation, the supernatants were kept and frozen. For serum isolation, whole blood was allowed to clot and supernatant (serum) was collected after centrifugation at 1,000×*g* for 15 min at +4°C. All samples were stored at −80°C. The IL-6 concentration was measured using mouse IL-6 Quantikine ELISA kit (R&D Systems, Minneapolis, MN, USA), according to manufacturer’s instructions.

### Detection of ROS Production and pSTAT3 Expression

Freshly isolated cells from BM or spleen of 12-week-old *N3-tg* and *wt* mice were obtained, and ROS production staining was performed upon incubation with MitoSOX Red 5 μM (Molecular Probes/Invitrogen/ThermoFisher, Waltham, MA, USA) for 30 min at 37°C. For the intracellular staining with anti-pSTAT3/pY705-PE (4/P-STAT3) antibody or relative isotype control (both from BD, Bioscience, La Jolla, CA, USA), BM cells were treated with Lyse Fix Buffer and Perm Buffer III (both from BD, Bioscience, La Jolla, CA, USA), according to the manufacturer’s recommendations, and then, samples were stained and processed for FACS analysis.

### Western Blotting

Total protein extracts were prepared from human cell line samples, and Western blotting analysis was conducted, as described elsewhere (28), using anti-Notch1-ICD Val1744 (D3B8) antibody (from Cell Signaling, Danvers, MA, USA). Anti-β-actin antibody (Sigma-Aldrich, St Louis, MA, USA) was used to normalize protein expression levels.

### Mice

Eight- to 12-week-old *N3-tg* mice (C57Bl/6 background) (9) and *wt* littermates were used. Immunodeficient nonobese diabetic/severe combined immunodeficient NOD.Cg-*Prkdc* ^scid^ *Il2rg* ^tm1Wjl^/SzJ (NSG) (29) mice were obtained from Charles River Laboratories (Calco, Italy). Mice were bred and housed in the Institute’s Animal Care Facilities. All mice were monitored daily and euthanized upon disease detection (9), as evidenced by an enlarged spleen, hunched posture, ruffled fur, reduced mobility, and/or labored breathing. Experimental groups were based on the age and genotype of mice. The number of mice used in each experiment was reported in figure legends. All procedures involving animals were approved by the local Animal Welfare Committee and conducted in accordance with the recommendations of the Italian national guidelines for experimental animal care and use (D.lgs 26/2014).

### *In Vitro* Suppression Assay and Induction of Murine MDSCs

*wt* T splenocytes were isolated by negative selection (PanT-Cell Isolation KitII, Miltenyi Biotec, Bergisch Gladbach, Germany) and stained with 2.5 μM CFSE (Sigma Aldrich, St Louis, MA, USA). CFSE-labeled T cells were activated with coated 3 μg/ml anti-CD3 and 2 μg/ml anti-CD28 (BD Bioscience , La Jolla, CA, USA), and 3.0 × 10^5^ cells/well (“responders”) were cocultured with graded numbers of CD11b^+^Gr1^+^ splenocytes (“suppressors”), sorted from the spleen of *wt* or *N3-tg* mice. Cocultures were performed for 72 h in 96-well plates. Samples were then harvested and stained, and proliferating cell percentage was evaluated as CFSE dilution by FACS analysis on gated CD4^−^CD8^+^ T cells. To induce MDSCs from BM myeloid progenitors (30), 3.5 × 10^6^/well of total cells from *wt* BM were placed in 6-well plates and cocultured for 5 days at a 1:1 ratio with sorted BM DP T cells from 12-week-old *N3-tg* mice by using transwell inserts (pore diameter, 0.4 μm, Falcon/Corning, NY, USA). The Gr1^+^ cells (suppressors) that were also CD11b^+^ at 99.0% (not shown) were then selected from the harvested samples (purity ≥93%) by using biotin-anti-Gr1 antibody and streptavidin magnetic microbeads (Miltenyi Biotec, Bergisch Gladbach, Germany) and were cultured with activated and CFSE-labeled *wt* T splenocytes (responders), to assess their function, as above. We used Gr1^+^ fractions from *wt* BM cells cultured alone or supplemented with GM-CSF 40 ng/ml (STEMCELL Technologies, Vancouver, Canada) as negative and positive controls. For IL-6 neutralization, we performed MDSC induction assay in the presence of anti-IL-6 (MP5-20F3) antibodies or isotype controls (BD Bioscience, La Jolla, CA, USA) at 2 μg/ml.

### Treatment of *N3-tg* Mice With Anti-IL-6 or Anti-Gr-1 Antibodies

To neutralize IL-6, we used 300 μg/mouse of anti-mouse IL-6 (MP5-20F3) antibodies or RatIgG anti-horseradish peroxidase (HRPN) isotype controls (both *in vivo*MAb from BioXCell, Lebanon, NH, USA). To deplete Gr-1^+^ cells, we used 250 μg/mouse of anti-mouse Ly6G/Ly6C (Gr-1, RB6-8C5 clone) antibodies or RatIgG2b (LTF-2 clone) isotype controls (both *in vivo*Plus from BioXCell, Lebanon, NH, USA). In another set of experiments, mice were treated with both anti-IL-6 and anti-Gr-1 antibodies. We treated 8-week-old *N3-tg* mice with intraperitoneal injections of the relevant antibodies, resuspended in 200 μl/mouse of PBS1×, twice a week for 4 weeks. Mice were then sacrificed and characterized by FACS analysis, as above. Some of the mice were also intraperitoneally injected with BrdU (1.5 mg/mouse; BD Bioscience, La Jolla, CA, USA), 24 h before the sacrifice. Single-cell suspensions from the spleen and BM were then stained with the BrdU-flow kit (BD Bioscience, La Jolla, CA, USA), according to the manufacturer’s instructions, and Gr-1^+^BrdU^+^ and CD4^+^CD8^+^BrdU^+^ cell percentages were assessed by FACS analysis. At the end of the assay, Gr-1^+^ cell fractions were magnetically purified from spleens and tested by suppression assay, as above.

### Adoptive Transfer of CD4^+^CD8^+^ (DP) T Cells

We intravenously injected 6–10-week-old NSG female mice with 2–4 × 10^6^ of DP T cells sorted from the BM of 8-week-old *N3-tg* mice or thymus of *wt* littermates and resuspended in 200 μl/mouse of PBS1×. Each of the *N3-tg* or *wt* donors had two NSG recipients that were sacrificed at 3–5- and 9–11-week posttransplantation, respectively, and analyzed by FACS procedures. CD11b^+^Gr-1^+^ BM cells from NSG recipients at 9–11-week posttransplantation were sorted and assessed by suppression assay, as above.

### Cell Lines

The human cell lines of Notch1-dependent T-ALL, KE-37 (11) and of Notch3-dependent T-ALL, TALL-1 (31), and the human Notch-independent T-ALL cell line, Loucy (12, 32), were cultured at 37°C and 5% CO_2_ in RPMI-1640 (GIBCO/ThermoFisher, Waltham, MA, USA) complete medium, supplemented with 10% FBS, 10 U/ml penicillin and streptomycin, and 2 mM glutamine. The Loucy cell line was purchased from the ATCC (#CRL-2629). Cells were routinely verified to be mycoplasma free by using a PCR detection kit (Mycoplasma #G238, Abm Inc., Richmond, Canada).

### Induction of Human MDSCs

Human PBMCs from healthy donors were isolated by density gradient centrifugation on Ficoll-PaquePLUS (GE Healthcare-Amersham, GB). We put 5 × 10^6^/well of PBMCs in 6-well plates and cocultured with 2.5 × 10^6^ of KE-37 or TALL-1 cell lines, in transwell inserts (pore diameter, 0.4 μm, Falcon/Corning, NY, USA), for 6 days. Autologous PBMCs cultured in medium alone were run in parallel, as a control. The T-ALL cells were then removed, and all the remaining cells were collected. Adherent cells were harvested by using the Accutase detachment solution (BD Bioscience, La Jolla, CA, USA). In some cases, the cocultures were conducted in the presence of neutralizing anti-IL-6 (MQ2-13A5) antibodies or isotype controls (BD Bioscience, La Jolla, CA, USA), at 10 μg/ml, or with GSI IX (DAPT, Calbiochem/Sigma Aldrich, St Louis, MA, USA), at 10 μM. In another set of experiments, GSIs and anti-IL-6 antibodies were used together. The CD33^+^ cell fractions were isolated from the harvested samples above, by using anti-CD33 magnetic microbeads (Miltenyi Biotec, Bergisch Gladbach, Germany; purity ≥92%), and evaluated by their ability to inhibit the proliferation of CD4^−^CD8^+^ T-gated cells from CFSE-stained autologous PBMCs, upon activation with coated anti-CD3 and soluble anti-CD28 (at 1 and 5 μg/ml, respectively, BD Bioscience, La Jolla, CA, USA), at 72 h of culture. Blood samples from healthy donors were obtained upon written informed consent, and all the human investigations were conducted in accordance with the Declaration of Helsinki, and approval was obtained from the Institutional Ethics Committee.

### Statistical Analysis

Data were reported as mean ± SD from at least three independent experiments unless otherwise specified. Statistical analysis was performed using unpaired 2-tailed Student’s *t*-test to analyze the difference between two groups, using GraphPad software (San Diego, CA, USA). *p*-values of ≤0.05 are considered statistically significant. ^*^ *p* ≤ 0.05, ^**^ *p* ≤ 0.01, ^***^ *p* ≤ 0.001, and ^****^ *p* ≤ 0.0001 are significant differences between the indicated groups; not significant (ns), *p* > 0.05.

## Results

### MDSCs Populate the T-ALL Environment of *N3-tg* Mice

In murine models of Notch-dependent T-ALL, aberrant immature T cells, including CD4^+^CD8^+^ (DP) T cells, infiltrate the BM (8, 10, 28, 33). Immature T cells from Notch-dependent T-ALL-bearing mice are also able to induce non-cell autonomous expansion of the myeloid compartment (25, 26). In particular, *N3-tg* mice develop an aggressive form of T-ALL. The typical disease signs (enlarged spleen, hunched posture, ruffled fur, reduced mobility, and/or labored breathing) become evident starting at 6–8 weeks of age and 95% of animals have died by 16 weeks of age (9). They develop tumors with heterogeneous phenotypes, regarding the expression of CD4 and CD8 markers, including some in which peripheral tumor T cells display a prevalent CD4^−^CD8^−^ or CD4^+^CD8^−^ or CD4^−^CD8^+^ phenotype (9). However, more than 90% of *N3-tg* transgenic mice show the presence of aberrant DP T cells in the BM and spleen (AFC and PG, unpublished data), and their accumulation in the periphery raises along with the disease progression and represents a pathognomonic feature (33–35).

**Figure 1:**
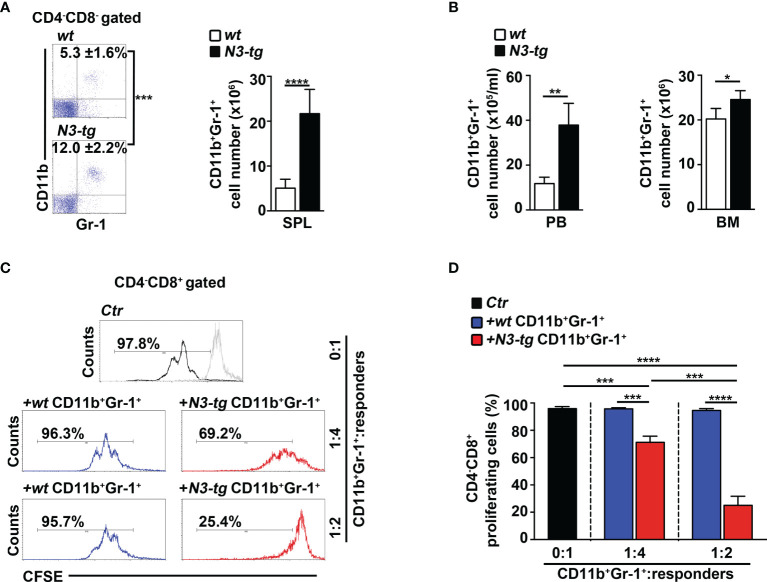
CD11b^+^Gr-1^+^ MDSCs expand in *N3-tg* mice. **(A)** Percentages and absolute numbers of CD11b^+^Gr-1^+^ cells, as assessed by FACS analysis distribution of CD4 vs. CD8 vs. CD11b vs Gr-1 markers in spleen (SPL) of *N3-tg* and *wt* mice. Numbers inside cytograms indicate mean percentages ± SD of CD11b^+^Gr-1^+^ cells. The cytograms in this panel illustrate the percentages of CD11b^+^Gr-1^+^ splenocytes inside the CD4^−^CD8^−^ subset, to avoid any possible dilution effect due to the presence of DP T cells in transgenic mice. **(B)** CD11b^+^Gr-1^+^ numbers in peripheral blood (PB) and BM from *N3-tg* vs. *wt* mice, assessed by FACS analysis, as in **(A)**. In **(A, B)**, the values are presented as mean ± SD of four independent experiments (*n* = 6 mice per group). **(C)** Representative FACS analysis of suppression assay with CFSE-labeled/activated *wt* T splenocytes (“responders”), cultured either alone (Ctr), as a control, or in combination with CD11b^+^Gr-1^+^ “putative” MDSCs (“suppressors”). Numbers inside cytograms represent percentages of proliferating CD4^−^CD8^+^ responder cells. Similar results were obtained by analyzing the proliferation rate of gated *wt* CD4^+^CD8^−^ T responder cells (not shown). CD11b^+^Gr-1^+^ cells were sorted from spleen of *N3-tg* or *wt* mice and used at the indicated CD11b^+^Gr-1^+^:responders ratio. The gray line represents nonactivated negative controls. In **(D)**, results of suppression test, as in **(C)**, are illustrated as mean values ± SD from three independent experiments (*n* = 3 mice per group), with two technical replicates per experiment. All the mice were analyzed at 12 weeks of age. ^*^ *p* ≤ 0.05, ^**^ *p* ≤ 0.01, ^***^ *p* ≤ 0.001, and ^****^ *p* ≤ 0.0001 represent significant differences between the indicated groups.

We have already shown that aberrant DP T cells massively colonize the BM of *N3-tg* mice and that their numbers increase with age (33). Now, we report a marked expansion of the CD11b^+^Gr-1^+^ myeloid subset in the spleen (**Figure 1A**), as well as in peripheral blood (PB) and BM (**Figure 1B**) of *N3-tg* mice, when compared with *wt* littermates.

In mice, CD11b^+^Gr-1^+^ MDSCs express a high level of “reactive species of oxygen” (ROS) and arginase-1, which are related to the immunosuppressive function (36), and STAT3-signaling activation is crucial during their expansion/activation phase (37). We found a significant increase of arginase-1 expression, at mRNA and protein levels (**Supplementary Figures S1A, B**, respectively), and of the proportion of both ROS-producing cells (**Supplementary Figure S1C**) and phosphorylated STAT3 (pSTAT3) protein-expressing cells (**Supplementary Figures S1D, E**), inside the Gr-1^+^ subset from *N3-tg* mice, with respect to *wt* counterparts. However, the immunosuppressive function remains one of the most important features of MDSCs (13). Strikingly, we observed that CD11b^+^Gr-1^+^ splenocytes from *N3-tg* mice do exert *in vitro* a dose-dependent suppressive activity on proliferating *wt* CD4^−^CD8^+^ T cells, which is significantly higher than that of *wt* controls (**Figures 1C, D**).

Overall, our results demonstrate that functional CD11b^+^Gr-1^+^ MDSCs are present in the tumor environment of Notch3-dependent T-ALL.

### CD4^+^CD8^+^ (DP) T Cells from *N3-tg* Mice Induce MDSCs *In Vitro*, Through an IL-6-Dependent Mechanism

**Figure 2:**
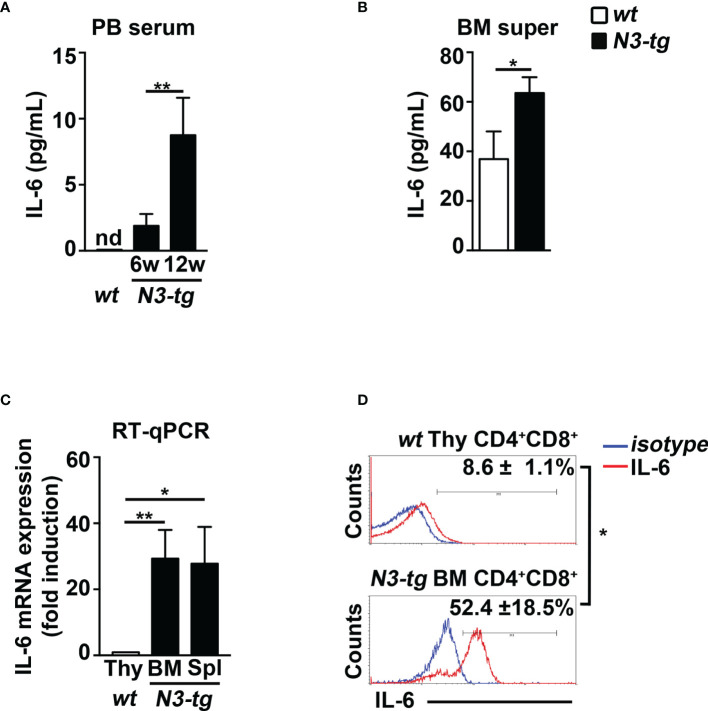
*N3-tg* mice display high levels of IL-6. IL-6 protein concentration assessed by ELISA **(A)** in blood serum (PB serum), from *N3-tg* vs. *wt* mice at 6 and 12 weeks of age, and **(B)** in BM supernatant (BM super), from *N3-tg* vs. *wt* mice at 12 weeks of age. **(C)** RT-qPCR of relative IL-6 mRNA expression in DP T cells sorted from BM or spleen (Spl) of *N3-tg* mice or from *wt* thymus (Thy), as a control, at 12 weeks of age. IL-6 mRNA expression levels in *wt* DP thymocytes are set as 1. **(D)** Representative FACS analysis of intracellular IL-6 staining in DP T cells from *N3-tg* BM or from *wt* thymus (Thy), at 12 weeks of age, upon 4 h of PMA/ionomycin stimulation (red lines). Blue lines, isotype controls. Numbers inside cytograms represent mean percentages ± SD of IL-6^+^ cells inside CD4^+^CD8^+^ subset. The values are presented as mean ± SD from three independent experiments (*n* = 4 mice per group), and in **(A–C)** with three technical replicates per experiment. nd, not detectable; ^*^ *p* ≤ 0.05 and ^**^p ≤ 0.01 represent significant differences between the indicated groups.

**Figure 3:**
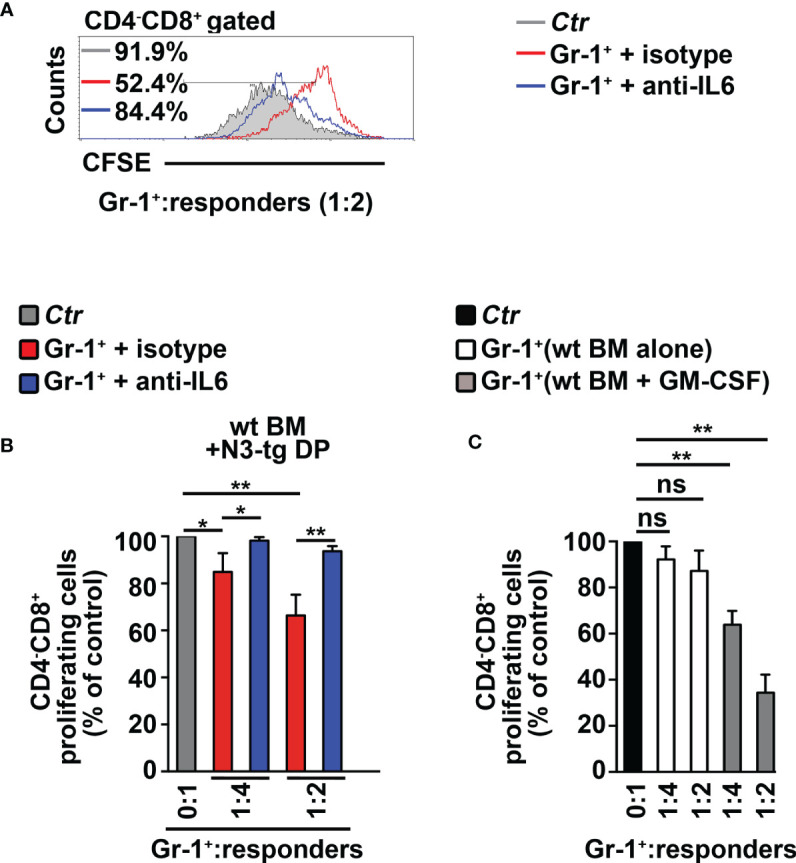
CD4^+^CD8^+^ (DP) T cells from *N3-tg* mice drive *in vitro* differentiation of MDSCs through IL-6. **(A)** Representative FACS analysis of suppression assay with CFSE/activated *wt* T splenocytes (“responders”), cultured either alone (Ctr), as a control, or in combination with Gr-1^+^ cells (Gr-1^+^), at the indicated Gr-1^+^/responders ratio. Numbers inside cytograms indicate the percentages of proliferating *wt* CD4^−^CD8^+^ T responder cells. Gr-1^+^ cells were magnetically selected from 5-day cocultures of total *wt* BM cells with DP T cells from BM of *N3-tg*, in the presence of anti-IL-6-neutralizing antibodies (blue line) or isotype controls (red line), and by using transwell inserts. In **(B)**, the results of the same suppression test, as in **(A)**, are illustrated as mean values ± SD from multiple experiments, see below. **(C)** The suppression test was performed by using *wt* T responder cells cultured either alone (black bar), as a control, or in combination with Gr-1^+^ cells (Gr-1^+^), selected, as above, from total *wt* BM cells cultured in medium alone or supplemented with GM-CSF (white bars and gray bars, respectively), for 5 days, and used at the indicated Gr-1^+^:responders ratios. In **(B, C)**, the results are calculated as the ratio between percentages of proliferating CD4^−^CD8^+^ T responder cells in Gr-1^+^-containing cultures and in controls, set up in the absence of Gr-1^+^ cells, and are expressed as % of control. The results represent the mean values ± SD from three independent experiments (*n* = 3 mice per group), with two technical replicates per experiment. ns, not significant, *p* > 0.05; ^*^ *p* ≤ 0.05, and ^**^ *p* ≤ 0.01 represent significant differences between the indicated groups.

To start shedding light on the mechanism of Notch-driven MDSC induction, we explored the potential role of the proinflammatory cytokine IL-6, a remarkable factor in the expansion/activation of MDSCs (37). The IL-6 concentration in blood serum is undetectable in *wt* controls, whereas it increases with age in *N3-tg* mice (**Figure 2A**). IL-6 concentration is also higher in the BM supernatants of *N3-tg* mice, compared with *wt* littermates (**Figure 2B**). Furthermore, DP T cells from transgenic animals express more IL-6 at mRNA level (**Figure 2C**) and present a greater percentage of IL-6 protein-expressing cells (**Figure 2D**) when compared with control *wt* DP thymocytes. We hypothesized that *N3-tg* DP T cells could contribute to inducing MDSCs through IL-6. It is important to note that literature shows that it is possible to drive *in vitro* the differentiation of MDSCs from their *wt* BM precursors, through their exposure to cytokines, including IL-6 (30), but also in coculture with tumor cell lines (38). Thus, we performed coculture experiments of total BM cells from *wt* mice with DP T cells from the BM of *N3-tg* mice, in the presence of neutralizing anti-IL-6 antibodies or isotype controls and by using transwell inserts. At the end of the assay, we selected Gr-1^+^ fractions from the harvested coculture samples and controls and used them in a suppression test. We observed that Gr-1^+^ cells selected from *wt* BM cocultured with *N3-tg* DP T cells are functional MDSCs, whereas inhibitory activity is greatly impaired by the addition of anti-IL-6 antibodies during the coculturing time (**Figures 3A, B**). The suppressive function of control Gr1^+^ cells selected from *wt* BM cultured in medium alone (**Figure 3C**, white bars), as well as in the presence of anti-IL-6 antibodies or isotype controls (**Supplementary Figure S2A**), was instead negligible. We also used Gr1^+^ cells selected from *wt BM* cells cultured in medium supplemented with GM-CSF, as positive controls (**Figure 3C**, grey bars). Furthermore, Gr1^+^ cells from *wt* BM cultured with recombinant IL-6 or cocultured with *wt* DP thymocytes, in the presence or not of IL-6, are not suppressive (**Supplementary Figure S2B**), suggesting that induction of MDSCs requires *N3-tg* DP T cells. As stated above, aberrant DP T cells colonize the periphery in the vast majority of *N3-tg* animals, and thus, we focused our observations and experiments on them. However, other T-cell subsets from *N3-tg* mice may include tumor cells and could exert similar effects on MDSCs. Indeed, *N3-tg* non-DP T cells (defined as a mixture of CD4^+^CD8^−^, CD4^−^CD8^+^, and CD4^−^CD8^−^ T cells, sorted from pre-enriched pan T-cell samples), as well as CD4^−^CD8^+^ cells from *N3-tg* mice are comparable with transgenic DP T cells in inducing MDSCs (**Supplementary Figure S2B**).

In summary, our data show that aberrant DP T cells from the BM of T-ALL-bearing *N3-tg* mice induce MDSCs, through a mechanism that is IL-6-dependent.

### CD4^+^CD8^+^ (DP) T Cells From *N3-tg* Mice Drive the Expansion of CD11b^+^Gr-1^+^ MDSCs in NSG Host Mice

**Figure 4:**
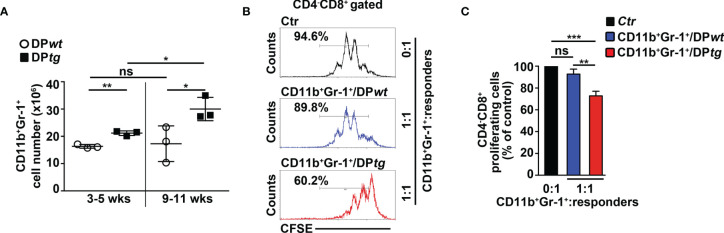
CD4^+^CD8^+^ (DP) T cells from *N3-tg* mice induce CD11b^+^Gr-1^+^ MDSCs in NSG hosts. NSG recipients were transplanted *i.v.* with CD4^+^CD8^+^ (DP) T cells sorted from BM of *N3-tg* mice (DP*tg*; *n* = 3 donors) or from thymus of *wt* mice (DP*wt*; *n* = 3 donors), as a control. Each of the donors has two recipients. NSG recipients were sacrificed at 3–5 weeks (*n* = 3 NSG recipients per group) and 9–11 weeks (*n* = 3 NSG recipients per group) posttransplantation and analyzed by FACS. **(A)** Absolute numbers of CD11b^+^Gr-1^+^ cells in BM of NSG mice, recipients of DP*wt* or DP*tg* cells, assessed by FACS analysis, at 3–5 and 9–11 weeks posttransplantation. **(B)** Representative FACS analysis of *in vitro* suppression assay of activated/CFSE-labeled *wt* T splenocytes (“responders”), cultured either alone (Ctr), as a control, or in combination with CD11b^+^Gr-1^+^ cells (as putative “suppressors”), at the indicated CD11b^+^Gr-1^+^/responders ratio. Numbers inside cytograms indicate the percentages of proliferating *wt* CD4^−^CD8^+^ T responder cells. CD11b^+^Gr-1^+^ cells were sorted from BM of NSG hosts, at 9–11 posttransplantation with DP*wt* (CD11b^+^Gr-1^+^/DP*wt*) or DP*tg* (CD11b^+^Gr-1^+^/DP*tg*) cells. In **(C)**, the results of suppression test, as in **(B)**, are calculated as ratio between percentages of proliferating *wt* CD4^−^CD8^+^ T cells in CD11b^+^Gr-1^+^-containing cultures (CD11b^+^Gr-1^+^/DP*wt* or CD11b^+^Gr-1^+^/DP*tg*) and in control cultures set up in the absence of CD11b^+^Gr-1^+^ (Ctr) and are expressed as % of control. Data represent the mean values ± SD from three independent experiments (*n* = 3 mice per group), with two technical replicates per experiment in **(C)**. ns, not significant, *p* > 0.05; ^*^ *p* ≤ 0.05, ^**^ *p* ≤ 0.01, and ^***^ *p* ≤ 0.001 represent significant differences between the indicated groups.

To corroborate *in vivo* our conclusions above, we performed adoptive transfer experiments of DP T cells from the BM of *N3-tg* mice or the thymus of *wt* mice, as a control. We used immunodeficient NSG hosts, which are devoid of T, B, and NK cells, but retain the CD11b^+^Gr-1^+^ subset (29). This model allows us to test if aberrant *N3-tg* DP T cells are able to drive the generation of MDSCs, by acting directly on their precursors, avoiding any possible interference by other immune-cell populations. We reported that *N3-tg* DP T cells (DP*tg*) efficiently engraft the BM of NSG recipients (**Supplementary Figure S3**), compared with what was observed with control *wt* DP thymocytes (*DPwt*). Notably, transplanted DP*tg* cells induce the expansion of the myeloid compartment in the BM of their NSG hosts; in fact, in the BM of these recipients, the CD11b^+^Gr-1^+^ absolute numbers progressively increase and are always significantly higher, with respect to those observed in NSG control mice, transplanted with DP*wt* control cells (**Figure 4A**). We then sorted CD11b^+^Gr-1^+^ cells from NSG-transplanted mice and tested them in an *in vitro* suppression test (**Figures 4B, C**). We observed that the CD11b^+^Gr-1^+^ cells from the NSG recipients of DP*tg* cells (CD11b^+^Gr-1^+^/DP*tg*) are functional MDSCs, whereas the suppressive ability of control counterparts (CD11b^+^Gr-1^+^/DP*wt*), is negligible. Overall, our data prove that N3-*tg* DP T cells can induce MDSCs *in vivo*. However, the NSG model has some limitations, and it will be interesting in the future to perform our adoptive transfer experiments in immunocompetent mice in order to conduct a more comprehensive analysis of T-ALL cell interactions with the immune system during MDSC induction.

### The Inhibition of IL-6 Impairs MDSCs in *N3-tg* Mice

**Figure 5:**
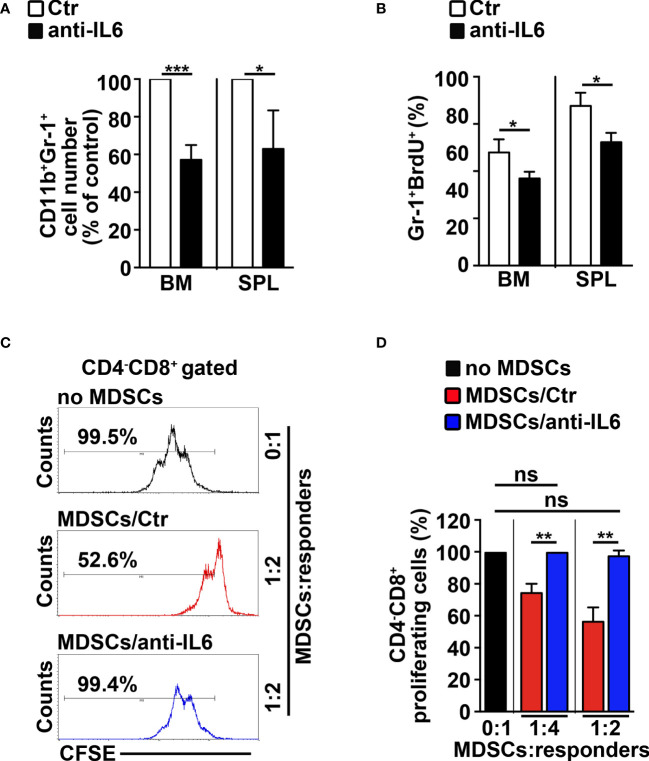
IL-6 neutralization impairs MDSCs in *N3-tg* mice. *N3-tg* mice were injected *i.p.* with neutralizing anti-IL-6 antibodies (anti-IL-6) or isotype controls (Ctr), twice a week and sacrificed/analyzed after 4 weeks of treatment; some of the mice were also injected *i.p.* with BrdU solution 1 day before the sacrifice. **(A)** The graph reports the ratio between the CD11b^+^Gr-1^+^ cell counts in anti-IL-6-treated *N3-tg* mice and in control mice, expressed as % of control, measured by FACS analysis in the BM and spleen (SPL) and presented as mean value ± SD from three independent experiments (*n* = 4 mice per group). **(B)** The percentages of Gr-1^+^BrdU1^+^ cells in the BM and SPL of anti-IL-6-treated *N3-tg* mice vs. controls are shown, assessed by FACS analysis. The data are presented as the mean value ± SD from two independent experiments (*n* = 3 mice per group). **(C)** Representative FACS analysis of suppression assay of CFSE-labeled/activated *wt* T splenocytes (“responders”), cultured either alone (no MDSC), as a control, or in combination with Gr-1^+^ MDSCs (“suppressors”), at the indicated MDSCs/responders ratio. Numbers inside cytograms indicate the percentages of proliferating *wt* CD4^−^CD8^+^ T responder cells. The Gr-1^+^ MDSCs were magnetically selected from spleen of *N3-tg* mice, treated with isotype control antibodies (MDSCs/Ctr) or anti-IL-6-neutralizing antibodies (MDSCs/anti-IL-6). In **(D)**, the results of the same suppression test, as in **(C)**, are illustrated as the percentage of proliferating CD4^−^CD8^+^ responder cells in MDSC-containing cultures (MDSCs/Ctr or MDSCs/anti-IL-6) and in control cultures set up in the absence of MDSCs (no MDSCs), at the indicated MDSCs:responders ratio. The results represent mean values ± SD from two independent experiments (*n* = 3 mice per group), with two technical replicates per experiment. ns, not significant, *p* > 0.05; ^*^ *p* ≤ 0.05; ^**^ *p* ≤ 0.01, ^***^ *p* ≤ 0.001 represent significant differences between the indicated groups.

To confirm the role of IL-6 in mediating the Notch-dependent induction of MDSCs, we treated *N3-tg* mice with repeated *i.p.* injections of neutralizing anti-IL-6 antibodies or isotype controls. The anti-IL-6-treated *N3-tg* mice reveal a significant decrease of CD11b^+^Gr-1^+^ cell count proportions (**Figure 5A**), and of the proliferating Gr-1^+^BrdU^+^ cell percentages (**Figure 5B**) in BM and spleen, when compared with controls, with no significant variation of total numbers of DP T- or other T-cell subsets (not shown), thus excluding that effects of neutralizing antibodies on Gr1^+^ subset are indirect. The *in vitro* suppression activity exerted by CD11b^+^Gr-1^+^ MDSCs from the spleen of *N3-tg* mice is impaired upon treatment with anti-IL-6 antibodies (**Figures 5C, D**). Thus, our *in vivo* experiments confirm the significant contribution of IL-6 in promoting the accumulation of MDSCs.

### MDSCs Sustain Expansion and Proliferation of CD4^+^CD8^+^ (DP) T Cells in *N3-tg* Mice

**Figure 6:**
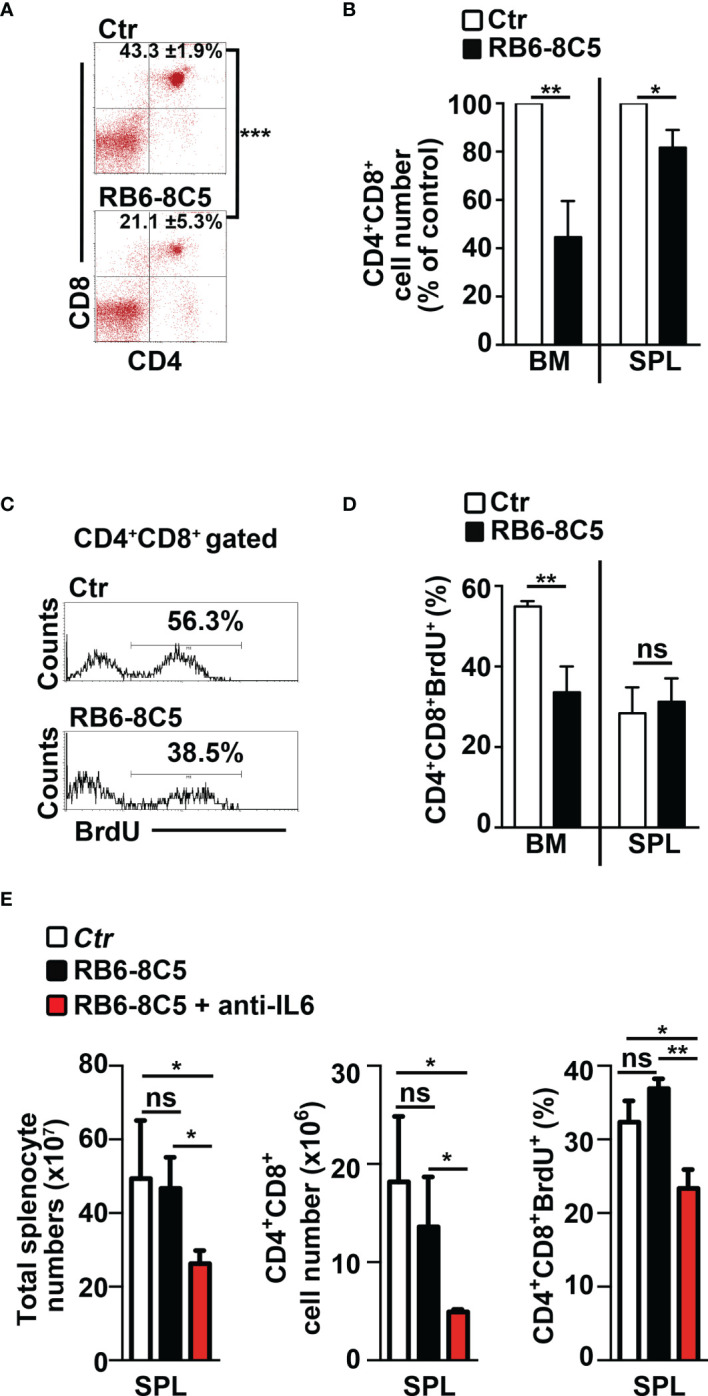
Gr-1 depletion affects CD4^+^CD8^+^ (DP) T cells in *N3-tg* mice. *N3-tg* mice were injected *i.p.* with anti-Gr-1 depleting antibodies (RB6-8C5) or isotype controls (Ctr), twice a week and sacrificed/analyzed after 4 weeks of treatment; some of the mice were also injected *i.p* with BrdU solution 1 day before the sacrifice. **(A)** Percentages of CD4^+^CD8^+^ cells are shown, as measured by FACS analysis in the BM of *N3-tg* mice, treated as above. Numbers inside cytograms indicate the mean percentages ± SD of CD4^+^CD8^+^ cells. **(B)** The graph reports the ratio between the CD4^+^CD8^+^ cell counts in RB6-8C5-treated *N3-tg* mice and in relative controls, expressed as % of control, and calculated in the BM and spleen (SPL), by the same FACS analysis, as in **(A)**. In **(A, B)**, the data are presented as the mean value ± SD from three independent experiments (*n* = 4 mice per group). **(C)** Representative FACS analysis of BrdU^+^ cells inside the CD4^+^CD8^+^ subset in the BM of *N3-tg* mice, treated as above, with isotype control antibodies (Ctr) or anti-Gr-1 depleting antibodies (RB6-8C5). Numbers inside cytograms indicate the percentages of CD4^+^CD8^+^BrdU^+^ cells. **(D)** The graphs show the CD4^+^CD8^+^BrdU^+^ cell percentages in the BM and spleen (SPL) of *N3-tg* mice, treated as above, and measured by the same FACS analysis, as in **(C)**. We have not observed any effects of anti-Gr-1 treatment on T-cell compartment of *wt* mice (not shown). **(E)** The graphs report total cell counts (left panel), absolute numbers of DP T cells (middle panel), and percentages of BrdU^+^DP T cells (right panel), in mice injected with RB6-8C5 antibodies alone (black bars), or in combination with anti-IL-6 antibodies (red bars), compared with controls (white bars). In **(D, E)**, the results are presented as the mean value ± SD from two independent experiments (*n* = 3 mice per group). ns, not significant, *p* > 0.05; ^*^ *p* ≤ 0.05; ^**^ *p* ≤ 0.01, and ^***^ *p* ≤ 0.001 represent significant differences between the indicated groups.

We aimed to study the impact of MDSCs on T-ALL cell behavior. Thus, we performed experiments of i.p. injections with the RB6-8C5 antibody (39) that significantly deplete Gr-1^+^ cells in spleen and peripheral blood (**Supplementary Figures S4A, B**) and impair their suppressive function in BM (**Supplementary Figure S4C**) of *N3-tg* mice. Notably, this treatment leads to a significant decrease of the percentages and cell count proportions of DP T cells (**Figures 6A, B**), as well as of the CD4^+^CD8^+^BrdU^+^ cell percentages (**Figures 6C, D**), in the BM of anti-Gr-1-treated *N3-tg* mice, when compared with controls, with more limited effects on DP T splenocytes. We also reported that IL-6 protein levels in PB serum of anti-Gr1 *N3-tg*-treated mice decrease (**Supplementary Figure S4D**). This could be related to the reduction of IL-6-producing DP T cells in treated mice. However, we cannot rule out that it also derives from the depletion of IL-6-producing MDSCs/myeloid cells (40, 41).

Finally, we tried to improve the effects of MDSC depletion on disease progression by treating *N3-tg* mice with both anti-Gr-1 and neutralizing anti-IL-6 antibodies. Notably, we observed significant reductions of important parameters of disease progression in our model (9, 33–35), such as total splenocyte and DP T-cell numbers, as well as of Brdu^+^ DP T-cell percentages, in the spleen of *double-treated* mice, when compared with RB6-8C5-treated mice or controls (**Figure 6E**). In summary, our results show that MDSCs sustain the accumulation and proliferation of aberrant DP T cells from Notch3-dependent T-ALL.

### Human Notch-Dependent T-ALL Cell Lines Induce MDSCs From Healthy PBMCs

**Figure 7:**
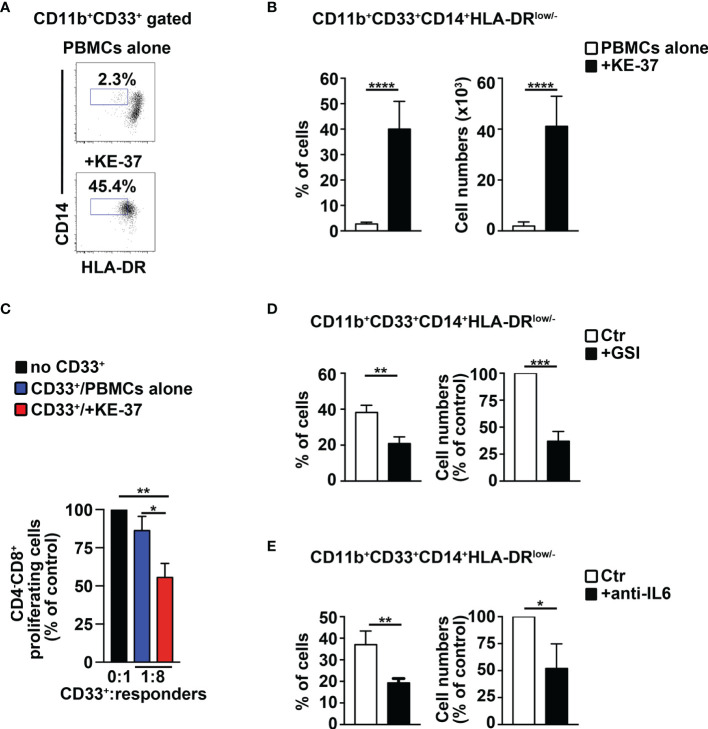
The Notch1-active T-ALL cell line, KE-37 induces MDSCs from healthy PBMCs. PBMCs from healthy donors were cocultured with the human Notch1-dependent T-ALL cell line, KE-37, or were cultured in medium alone, as a control, for 6 days, by using transwell inserts. The data shown are relative to the end of the culture. **(A)** Representative dot plot of CD14^+^HLA-DR^low/neg^ cells inside CD11b^+^CD33^+^ subset, as measured by FACS analysis in harvested PBMCs/KE-37 coculture samples (+KE-37), *versus* samples of autologous PBMCs cultured alone (PBMCs alone). Numbers inside cytograms indicate the percentages of CD11b^+^CD33^+^CD14^+^HLA-DR^low/neg^ cells. **(B)** Percentages and absolute numbers of CD11b^+^CD33^+^CD14^+^HLA-DR^low/neg^ cells, measured by the same FACS analysis, as in **(A)**. The values are presented as mean ± SD from four independent experiments (*n* = 6 samples per group, donors *n* = 6), each in triplicates. **(C)** At the end of the coculture assay above, CD33^+^ fractions were magnetically selected from harvested samples and used as “putative” suppressors (CD33^+^) in a suppression test on proliferating CD4^−^CD8^+^-gated cells (“responders”), from CFSE-labeled/activated autologous PBMCs, at the indicated CD33^+^:responder ratios. CD33^+^ cells were selected from PBMCs cultured either in medium alone (CD33^+^/PBMCs alone) or with KE-37 cells (CD33^+^/+KE-37). The results are illustrated as the ratio between the percentages of proliferating CD4^−^CD8^+^-gated cells in cultures containing CD33^+^ and in control cultures set up in the absence of CD33^+^ (no CD33^+^) and expressed as % of control. In **(D, E)**, the graphs report percentages and cell count proportions of CD11b^+^CD33^+^CD14^+^HLA-DR^low/neg^ cells in harvested PBMCs/KE-37 cocultures, assessed by the same FACS analysis, as in **(A)**. The samples in **(D)** were cultured in the absence (CTR) or presence of GSI (+GSI), and the samples in **(E)** were cultured in the absence (CTR) or presence of anti-IL-6-neutralizing antibodies (+anti-IL-6). The cell count proportions were calculated as the ratio between the CD11b^+^CD33^+^CD14^+^HLA-DR^low/neg^ absolute numbers in PBMCs/KE-37 treated cocultures and in relative untreated controls and expressed as % of control. In **(C–E)**, the values represent the mean ± SD from three independent experiments (*n* = 3 samples per group, donors *n* = 3), each in triplicates. ^*^ *p* ≤ 0.05, ^**^ *p* ≤ 0.01, ^***^ *p* ≤ 0.001, and ^****^ *p* ≤ 0.0001 represent significant differences between the indicated groups.

We tried to extend our remarks above to humans, by performing coculture experiments of PBMCs from healthy donors with human Notch-dependent T-ALL cell lines. First, we used the Notch1-activated KE-37 cell line, being Notch1 a frequent target of oncogenic gain-of-function mutations in T-ALL patients (11). Interestingly, KE-37 cells are GSI resistant and Notch3 negative (42) and express intracellular IL-6 (**Supplementary Figure S5A**). The harvested PBMCs/KE-37 coculture samples reveal a significant increase of CD14^+^HLA-DR^low/neg^ “putative” MDSCs, both in percentages (**Figures 7A, B**, left panel) and absolute numbers (**Figure 7B**, right panel), when compared with samples of autologous PBMCs cultured alone. At the end of the culture, we selected the CD33^+^ fractions from all the harvested samples and assessed their suppressive function on autologous CD4^−^CD8^+^ T cells. We revealed that CD33^+^ cells from PBMCs/KE-37 cocultures are highly suppressive MDSCs (**Figure 7C**) when compared with those from PBMCs cultured alone. Importantly, the KE-37 line is part of a group of human T-ALL cell lines in which the growth is not inhibited by GSI treatment, probably due to a lack in the expression of the *PTEN* gene, although GSI is effective in blocking Notch1 activation [(43) and references therein]. Indeed, we confirmed the expression of Notch1 intracellular domain in KE-37 cells and its downmodulation upon GSI treatment, during cocultures with PBMCs (**Supplementary Figure S6A**). We then repeated the PBMCs/KE-37 coculture experiments in the presence of gamma-secretase inhibitors or neutralizing anti-IL-6 antibodies. We observed that the inhibition of Notch activation or the block of IL-6 signals induces significant reductions of MDSC expansion (**Figures 7D, E**, respectively).

We measured the expression of IL-6 protein by FACS analysis in T-ALL cells, as well as in cell subsets of human PBMCs in our experiments of cocultures. At day 6, KE-37 cells produce IL-6 when cultured alone and also upon cocultures with PBMCs, showing no significant variation between the two conditions (**Supplementary Figure S6B**, left panel). Conversely, the expression of IL-6 appears very low in both T-cell and myeloid cell compartments of PBMCs cultured either alone or with KE-37 line (**Supplementary Figure S6B**, middle and right panels, respectively). Our results suggest that T-ALL cells may represent the main producer of IL-6 during MDSC induction, at least *in vitro*.

Furthermore, we performed experiments above with the Notch3-activated T-ALL cell line, TALL-1 (31) that is IL-6^+^ (**Supplementary Figure S5A**) and Notch1-negative (42). TALL-1 cells promote the induction of suppressive MDSCs (**Supplementary Figures S5B–D**) that is inhibited by exposure to GSIs or anti-IL-6 antibodies (**Supplementary Figures S5E, F**, respectively). Collectively, our results indicate that Notch3 deregulation, as well as Notch1 deregulation, is capable of inducing MDSCs. In other words, it seems that the activation of Notch pathway in a T-ALL context induces MDSCs no matter which Notch receptor is activated.

We then tried to better clarify the Notch/IL-6 crosstalk during the induction of MDSCs, by using both GSIs and neutralizing anti-IL-6 antibodies, compared with GSIs alone, in our coculture assay of healthy PBMCs with TALL-1 cells. We have observed no significant difference between the two treatment conditions regarding the inhibition of MDSC induction (**Supplementary Figure S5G**); similar results were obtained with PBMCs/KE-37 cocultures (not shown). These data sustain the hypothesis that IL-6 cooperates with Notch-signaling activation in inducing MDSCs, but the last one represents the necessary condition to observe this induction.

**Figure 8:**
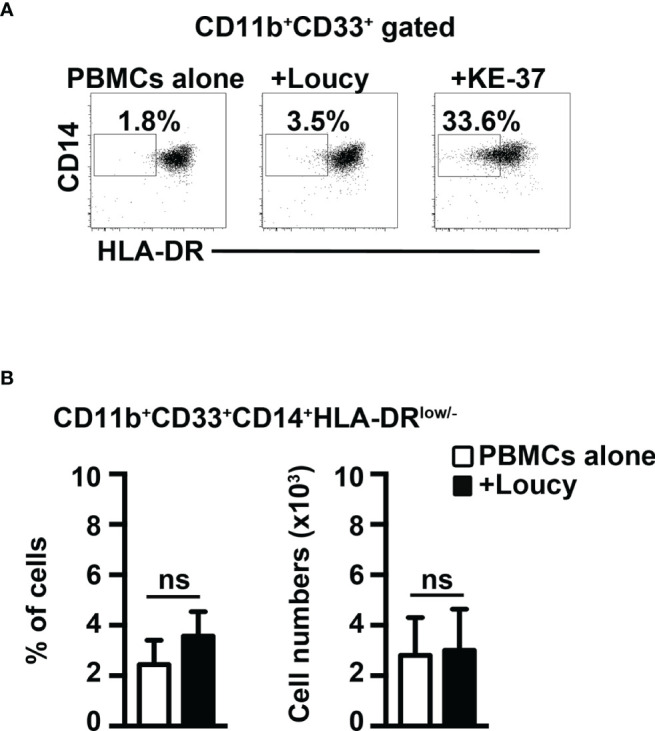
The Notch-inactive T-ALL cell line, Loucy, does not induce MDSCs from healthy PBMCs. **(A)** Representative dot plot of CD14^+^HLA-DR^low/neg^ cells inside CD11b^+^CD33^+^ subset, as measured by FACS analysis in harvested PBMCs/Loucy coculture samples (+Loucy), vs. samples of autologous PBMCs cultured alone (PBMCs alone). PBMCs/KE-37 coculture samples (+KE-37) were also included as positive controls of MDSC induction. Numbers inside cytograms indicate the percentages of CD11b^+^CD33^+^CD14^+^HLA-DR^low/neg^ cells. **(B)** Percentages (left panel) and absolute numbers (right panel) of CD11b^+^CD33^+^CD14^+^HLA-DR^low/neg^ cells, as measured by the same FACS analysis, as in **(A)**. In **(B)**, the values represent the mean ± SD from three independent experiments (*n* = 4 samples per group, donors *n* = 4), each in duplicates. ns, not significant.

Finally, we performed coculture experiments of healthy PBMCs with the human T-ALL cell line, Loucy, that does not express Notch1- or Notch3-activated protein (12, 32). Notably, we have not observed any evident induction of MDSCs (**Figure 8**). In summary, we show that Notch-dependent T-ALL cells promote human MDSCs *in vitro*, through a mechanism that depends on both Notch and IL-6, consistently with findings in our mouse model.

## Discussion

T-ALL accounts for 10%–15% of pediatric and 25% of adult ALL cases. Despite the efficacy of current chemotherapy protocols, 25%–30% of children and up to 60% of adults among T-ALL patients still relapse (44, 45). Bone marrow is the most common site of T-ALL recurrence, and BM relapse often represents a feature of worse prognosis (46, 47). Similarly, the BM of murine models of Notch-dependent T-ALL reveals precocious invasion of immature T cells (8, 10, 28, 33) that perturb stromal niches and hematopoiesis (25, 26). Here, we report that aberrant T cells from Notch3-dependent T-ALL participate in shaping the leukemia environment, through the control exerted on MDSCs. Indeed, we observed that CD4^+^CD8^+^(DP) T cells from the bone marrow of our murine model of Notch3-dependent T-ALL induce functional MDSCs *in vitro*, as well as in immunodeficient hosts.

Regarding the mechanism of MDSC induction, it has been well established that the development/activation of MDSCs depends on the production/release of proinflammatory cytokines, such as IL-6 (37). IL-6 expression is elevated in tumor cell lines that induce MDSCs (30) and in plasma of cancer patients, where its concentration positively correlates with the level of circulating MDSCs (48). In many tumors, including T-ALL, Notch signaling promotes IL-6 production by stromal components (26, 49). However, malignant cells themselves may represent the source of IL-6 (50, 51). We observed that IL-6 concentration is very high in blood serum and BM supernatant of *N3-tg* mice and that transgenic DP T cells express considerable levels of IL-6. Moreover, we reported that MDSC induction is significantly attenuated *via* neutralizing anti-IL-6 antibody exposure, *in vitro* and *in vivo*. These results emphasize the hypothesis that Notch-signaling deregulation in the T-cell compartment of T-ALL could contribute to inducing MDSCs, through an IL-6-dependent pathway. The use of transwell inserts in our *in vitro* coculture experiments supports a prevalent role of diffusible factors in Notch-driven induction of MDSCs. However, we cannot exclude that *in vivo* Notch activation may promote MDSCs, through other mechanism/s, such as those based on ligand/receptor interactions (23), and that other cell subsets may participate in releasing IL-6 in the tumor microenvironment.

Our *in vivo* Gr-1 depletion experiments suggest that MDSCs from *N3-tg* T-ALL mice limit significantly the expansion and proliferation of aberrant DP T cells, and then, they may eventually affect the disease outcome. Interestingly, by treating *N3-tg* mice with both anti-Gr1 and neutralizing anti-IL-6 antibodies, we observed a significant reduction of parameters that specifically mark the disease progression in our model, such as total numbers of splenocytes and splenic DP T-cell numbers (9, 33–35).

We need more efforts to elucidate the target/s and precise mechanism/s of MDSC action in T-ALL. However, summarizing our *in vivo* data, we can suggest some hypotheses (**Supplementary Figure S7**): it is very likely that alternative pathways are involved, besides the “classical” suppression of T-cell antitumor responses. MDSCs may act through the *de novo* generation of Tregs (36), including in cancer settings (52, 53), and Treg expansion has been described in the BM of T-ALL patients (54). In this regard, we demonstrated that Notch3 activation improves the generation and suppressive function of Tregs in *N3-tg* mice (55–57). MDSCs could also impinge on pathways that are underinvestigated in hematological malignancies, to date. They may target “innate” subsets engaged in cancer immune responses, such as NK cells (58), and/or exploit suppression *via* alternative signalings, such as the PD-1/PDL-1 axis (59, 60), with a potential impact on cancer immunotherapy (61, 62). In line with our hypothesis, a recent paper reports that myeloid cells can directly support T-ALL cell survival, independently from suppression of T-cell response (27). Thus, MDSCs may sustain T-ALL cell proliferation and/or survival and, eventually, tumor progression, either directly and, more likely, indirectly by blocking antitumor immune responses of NKs, besides that of CTL/Th1 subsets, as well as through the generation of immunosuppressive Tregs.

We start extending our preclinical observations to humans. We demonstrated that human Notch-dependent T-ALL cell lines sustain the expansion of functional MDSCs from normal PBMCs, in a coculture assay. Interestingly, by the exposure of cocultures above to GSIs or neutralizing anti-IL-6 antibodies or both, we suggested that Notch and IL-6 are both able to influence the MDSC induction; however, Notch-signaling activation remains the crucial event in this process, at least in our *in vitro* model with human T-ALLs.

Notably, the induction of MDSCs is not observed using a model of Notch-independent T-ALL, such as the Loucy cell line, suggesting that this process is specific for Notch-dependent T-ALL. Moreover, both Notch1 and Notch3 deregulation seems able to generate MDSCs from human PBMCs, as suggested by our observations on MDSC induction with human T-ALL cell lines, KE-37 and TALL-1, that overexpress in a mutually exclusive way active-Notch1 and active-Notch3, respectively. Notch1 has a prominent role in T-ALL development, and its activation by gain-of-function mutations has been reported in more than the 60% of T-ALL patients (11). Notch3 is often assumed as a target of Notch1 [ (63), and references therein], and it has been shown that Notch1 and Notch3 have common oncogenomic programs (64). On the other hand, aberrant Notch3 deregulation occurs in T-ALL cases lacking Notch1 activation (12, 63), and then, a specific Notch3-dependent subset of T-ALL patients may exist. Thus, we can speculate that, whatever the case, our preclinical conclusions in *N3-tg* mice, together with observations in humans, could have clinical relevance. It is important to note that, in the retroviral-vector-induced murine model of Notch1-dependent T-ALL, the expansion of CD11b^+^Gr-1^+^ myeloid cells was described in non-transduced populations (25, 26), and Notch3 was reported as a transcriptional target of Notch1 in transduced DP T cells from the BM (10). Based on these premises, it is likely that also in murine models of T-ALL, Notch1- and Notch3-signaling deregulation converge on the final effect of inducing MDSCs. However, this point deserves confirmation and detailed examination mainly focused on the mechanism/s involved. Remarkably, human T-ALL originates in the thymus and then infiltrates into the BM and peripheral blood [ (65) and references therein], and these features are well reproduced in the *N3-tg* model (9), which is based on the genetic alteration of this pathway at the level of immature thymocytes. In the Notch1 murine model, instead, deregulation of the Notch1 signal is already active in the BM where it is able to sustain T-cell development until the stage of DP T cells (8, 10).

Overall, we believe that our results represent the necessary premise for the analysis of MDSCs in T-ALL patients, with the final aim of defining them as a new target of therapy and/or as an innovative prognostic biomarker. Moreover, the *N3-tg* model could serve as a proof-of-concept system to deepen the studies on the bidirectional crosstalk between leukemic T cells, MDSCs, and other cell subsets, inside the T-ALL environment.

## Data Availability Statement

The raw data supporting the conclusions of this article will be made available by the authors, without undue reservation.

## Ethics Statement

The studies involving human participants were reviewed and approved by the Institutional Ethics Committee. The patients/participants provided their written informed consent to participate in this study. The animal study was reviewed and approved by the local animal welfare committee and conducted in accordance with the recommendations of the Italian national guidelines for experimental animal care and use (D.lgs 26/2014).

## Author Contributions

PG and AO designed and performed research, analyzed the data, and wrote the first draft of the paper. NG, CN, and BA performed the research. GP analyzed the data. IS critically revised the manuscript. AC supervised the experiments, analyzed the data, and wrote the manuscript. All authors listed have made a substantial, direct, and intellectual contribution to the work and approved it for publication.

## Funding

This work was supported by the Italian Association for Cancer Research (AIRC, IG13214) to IS, the FIRB Program (RBAP11WCRZ) to IS, the Sapienza University grants PH118164340087CF to IS and RM11715C7C626BAB to AC and by the Italian Ministry of Education, University and Research - Dipartimenti di Eccellenza - L. 232/2016.

## Conflict of Interest

The authors declare that the research was conducted in the absence of any commercial or financial relationships that could be construed as a potential conflict of interest.

## Publisher’s Note

All claims expressed in this article are solely those of the authors and do not necessarily represent those of their affiliated organizations, or those of the publisher, the editors and the reviewers. Any product that may be evaluated in this article, or claim that may be made by its manufacturer, is not guaranteed or endorsed by the publisher.
