# Supplementary material for: Notch-Signaling Deregulation Induces Myeloid-Derived Suppressor Cells in T-Cell Acute Lymphoblastic Leukemia
Source: Front Immunol. 2022 Apr 4;13:809261. doi: 10.3389/fimmu.2022.809261 (PMC9013886; doi:10.3389/fimmu.2022.809261)
Supplement: Supplementary file 1 [file DataSheet_1.docx]

***SUPPLEMENTARY MATERIAL***

**Supplementary Figures**


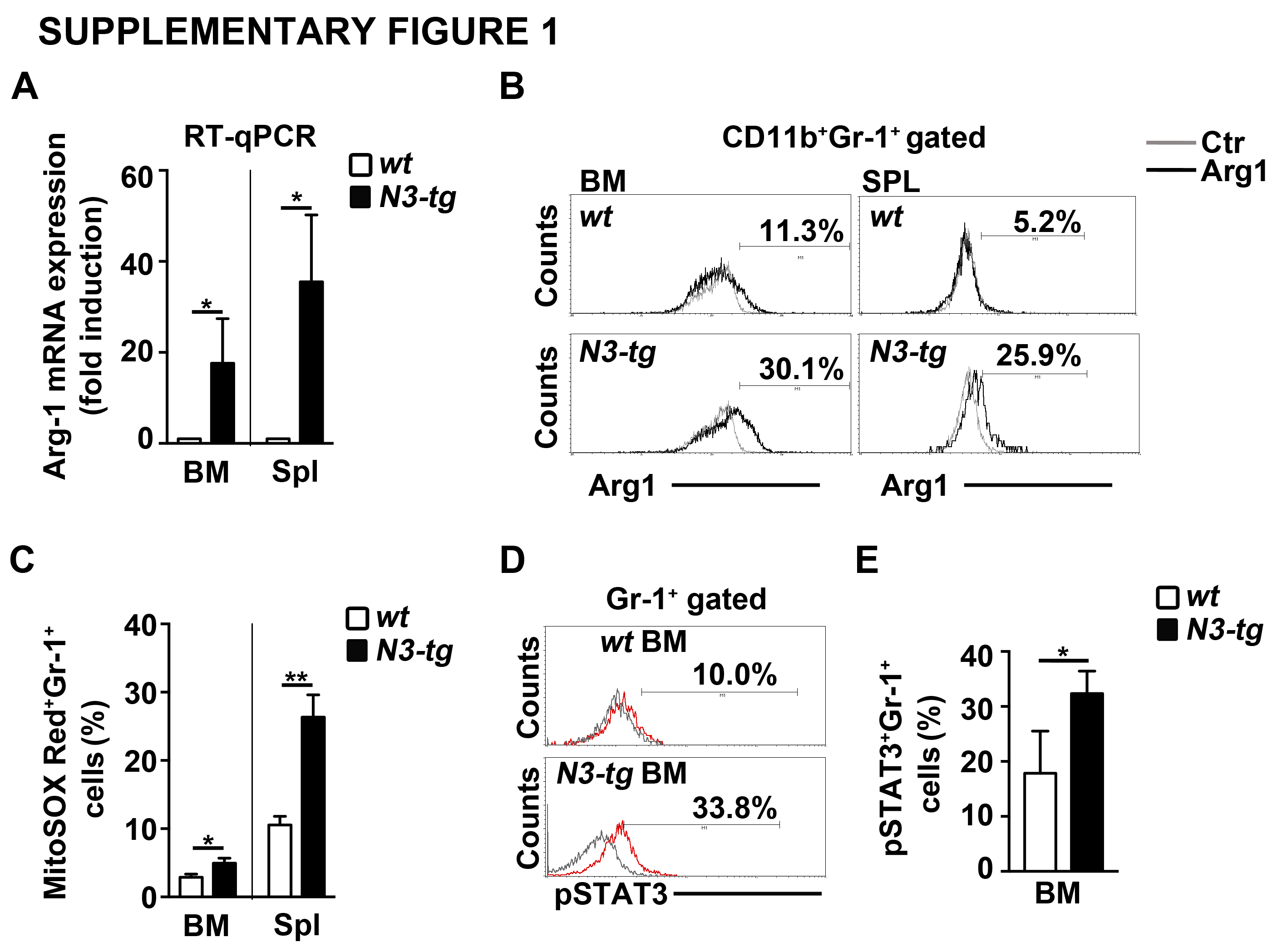


**Supplementary Figure 1. Gr-1+ cells from *N3-tg* mice display features of MDSCs.** **(A)** RT-qPCR assay, in duplicates, of relative Arginase-1 (Arg-1) mRNA expression in CD11b^+^Gr-1^+^ cells sorted from the bone-marrow (BM) or spleen (Spl) of *N3-tg versus wt* mice at 12 wks of age. The expression levels of Arg-1 mRNA in *wt* CD11b^+^Gr-1^+^ cells are set as 1. **(B)** FACS analysis of intracellular Arginase-1 protein expression reported as fluorescence intensity in CD11b^+^Gr-1^+^ gated cells from the bone-marrow (BM) or spleen (Spl) of *N3-tg versus wt* mice at 12 wks of age. Numbers inside cytograms indicate percentages of Arg-1^+^ cells (black line=anti-Arg-1; grey line=isotype ctr). Results are representative of three independent experiments (n=3 mice per group). **(C)** Percentages of MitoSOXRed^+^ cells inside Gr-1^+^ subset from BM or Spl of *N3-tg* at 12 wks of age, compared to *wt* littermates, as assessed by FACS analysis. In (A) and (B) the values are presented as mean±SD from three independent experiments (n=3 mice per group); *, P**≤**0.05 and **, P**≤**0.01 represent significant differences between the indicated groups. **(D)** Representative FACS analysis of pSTAT3 protein expression reported as fluorescence intensity in Gr-1^+^ gated cells from BM of *N3-tg* at 12 wks of age, compared to *wt* controls. Numbers inside cytograms indicate percentages of pSTAT3^+^ cells (red line=anti-pSTAT3; gray line=isotype ctr). **(E)** Percentages of pSTAT3^+^ cells in Gr-1^+^ gated cells from BM of *N3-tg* mice at 12 wks of age, compared to *wt* controls, as measured by the FACS analysis, as in (C). The values are presented as mean±SD from three independent experiments (n=3 mice per group); *, P**≤**0.05 represents significant differences between the indicated groups.

**
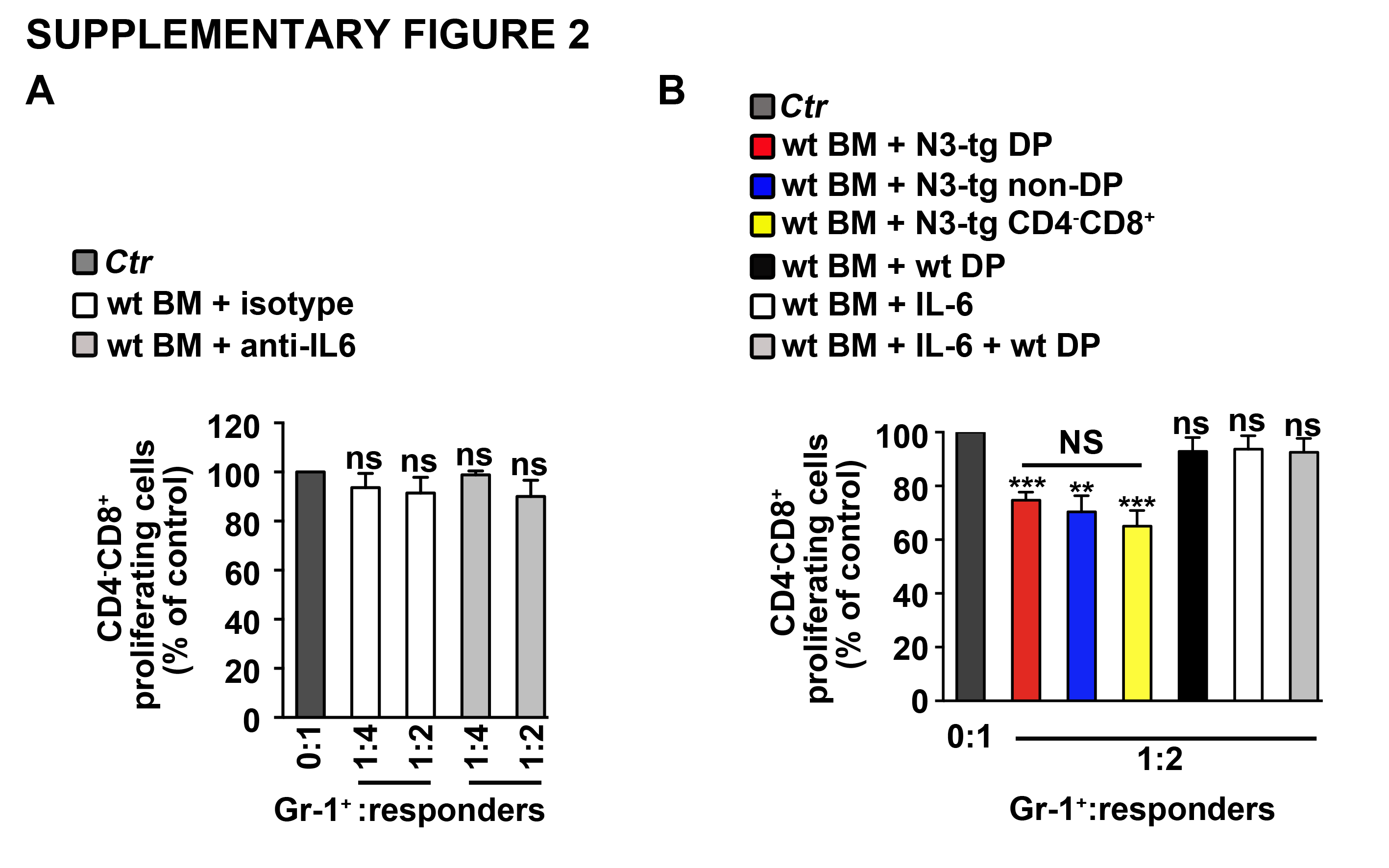
**

**Supplementary Figure 2. *N3-tg* T-ALL cells are required to generate functional MDSCs *in vitro* (A)** The graph represents the results of a suppression assay, indicating the percentages of proliferating *wt* CD4^-^CD8^+^ T 'responder' cells, assessed as CFSE dilution by FACS analysis, at 72 hrs of culturing, either alone (Ctr, dark grey bar), as a control, or in combination with Gr-1^+^ cells that were magnetically selected from five-days cultures of total *wt* BM cells, in the presence of anti-IL6 neutralizing antibodies (light grey bars) or isotype controls (white bars), at the indicated *wt* Gr-1^+^/responders ratio. **(B)** The same suppression test, as in (A), was conducted with *wt* Gr-1^+^ cells that were magnetically selected from five-days co-cultures, in the presence of transwells, of total *wt* BM cells with splenic *N3-tg* DP T cells, as a positive control (red bar); or with *N3-tg* non-DP T cells (blue bar); or with *N3-tg* CD4^-^CD8^+^ T cells (yellow bar); or with sorted *wt* DP thymocytes, either alone (black bar) or in the presence of mouse recombinant IL-6 (at a final concentration of 50 pg/mL, light grey bar); or, finally, cultured in the presence of IL-6 in the culture medium (white bar). In (A) and (B) the results in the graphs are calculated as the ratio between the percentages of proliferating CD4^-^CD8^+^ T ‘responder’ cells in Gr-1^+^ cells-containing cultures and in control cultures, set up in the absence of Gr-1^+^ cells, and are expressed as % of control. The results represent the mean values±SD from three independent experiments (n=3 mice per group), with two technical replicates per experiment. ns=not significant, P>0.05; **, P≤0.01 and ***, P≤ 0.001 represent significant differences with respect to the relative controls (Ctr). The NS symbol over the line represent not significant (P>0.05) differences between the indicated groups.

**
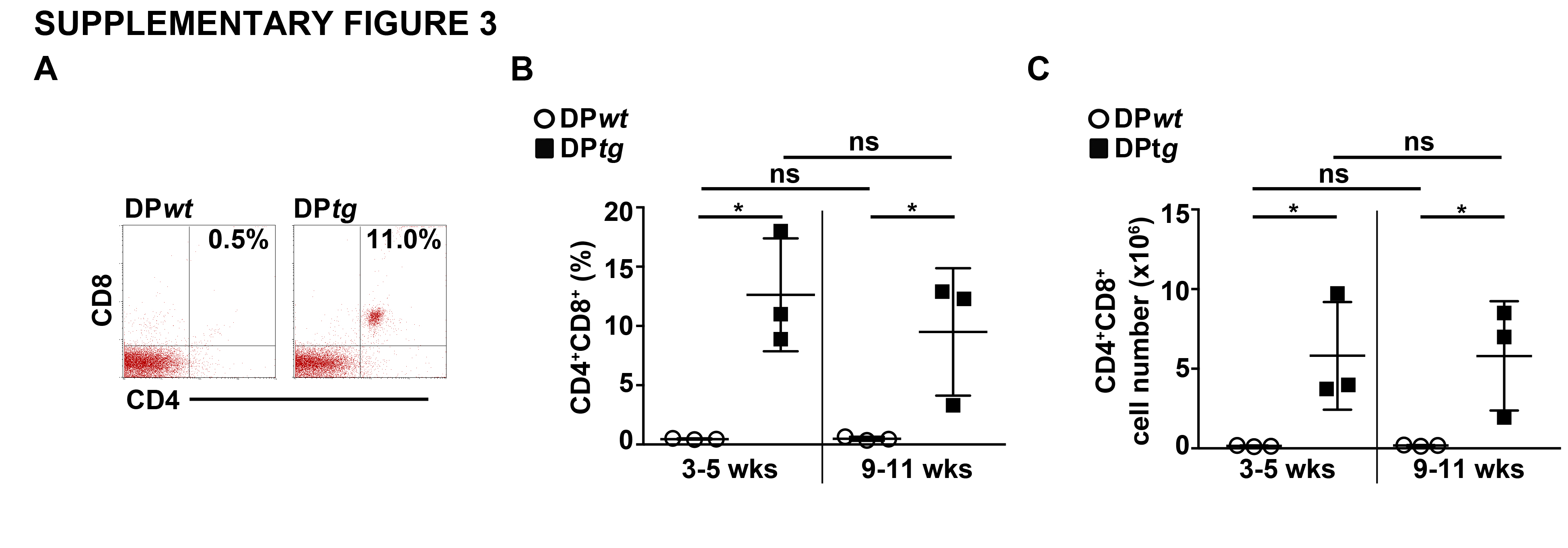
**

**Supplementary Figure 3. CD4^+^CD8^+^ (DP) T cells from *N3-tg* mice engraft the BM of NSG hosts.** The analysis presented in this figure were performed on the same NSG recipient groups, as in the Figure 4. **(A)** Representative FACS analysis of CD4 *versus* CD8 marker distributions in the BM of NSG mice, recipients of DP*tg* cells or DP*wt* control cells, at 3-5 wks post-transplantation. Numbers inside cytograms indicate the percentages of CD4^+^CD8^+^ cells. **(B)** Percentages and **(C)** absolute numbers of DP*tg* cells or DP*wt* control cells in the BM of their respective NSG hosts, at 3-5 wks and 9-11 wks post-transplantation, assessed by the same FACS analysis, as in (A). In panel (B-C) data are presented as the mean values±SD of three independent experiments (n=3 mice per group); ns=not significant, P>0.05; *, P≤0.05 represents a significant difference between the indicated groups.

**
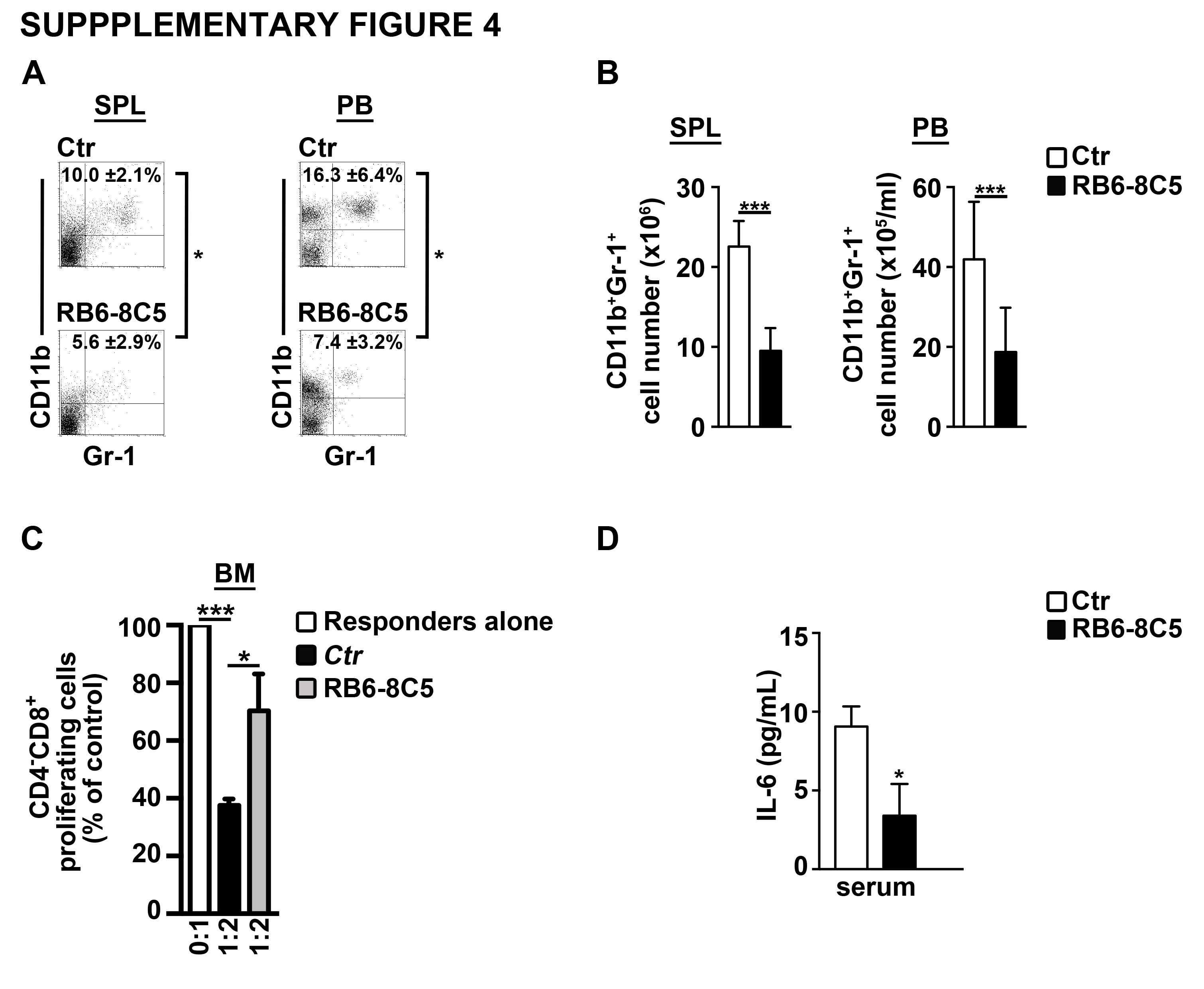
**

**Supplementary Figure 4. Treatment with RB6-8C5 antibodies impairs Gr-1^+^ subset of *N3-tg* mice.** *N3-tg* mice at 8 wks of age were injected *i.p.* with anti-Gr-1 (RB6-8C5 clone) depleting antibodies (RB6-8C5) or isotype controls (Ctr), twice a week and sacrificed and analyzed after 4 wks of treatment. **(A)** Percentages of CD11b^+^Gr-1^+^ cells are shown, as measured by FACS analysis of CD11b *versus* Gr-1 marker distributions in the spleen (SPL, left panel) and peripheral blood (PB, right panel) of *N3-tg* mice, treated as above. Numbers inside cytograms indicate the mean percentages±SD of CD11b^+^Gr-1^+^ cells. **(B)** The graphs show the CD11b^+^Gr-1^+^ absolute numbers in the spleen (SPL, left panel) and peripheral blood (PB, right panel) of *N3-tg* mice, treated as above, assessed by the same FACS analysis, as in (A). In (A) and (B) the values are presented as mean±SD of three independent experiments (n=4 mice per group). *, P≤0.05 and ***, P≤0.001 represent significant differences between the indicated groups. **(C)** The graph represents the results of a suppression assay, indicating the percentages of proliferating *wt* CD4-CD8+ T 'responder' cells, assessed as CFSE dilution by FACS analysis, at 72 hrs of culturing, either alone (Responders alone, white bar), or in combination with *N3-tg* Gr-1^+^ cells that were magnetically selected from BM of *N3-tg* mice, treated with RB6-8C5 antibodies (grey bar) or relative control antibodies (Ctr, black bar), as above. The indicated *N3-tg* Gr-1^+^/responders ratio (0:1, 1:2) were used. The results are calculated as the ratio between the percentages of proliferating CD4^-^CD8^+^ T ‘responder’ cells in Gr-1^+^ cells-containing cultures and in control cultures, set up in the absence of Gr-1^+^ cells, and are expressed as % of control. **(D)** IL-6 protein concentration assessed by ELISA in blood serum (PB serum), from *N3-tg* mice treated with RB6-8C5 *versus* relative controls (Ctr). In (C) and (D) the results are presented as the mean value±SD from two independent experiments (n=3 mice per group), and with two technical replicates per experiment. *, P≤0.05 and ***, P≤0.001 represents significant differences between the indicated groups.

**
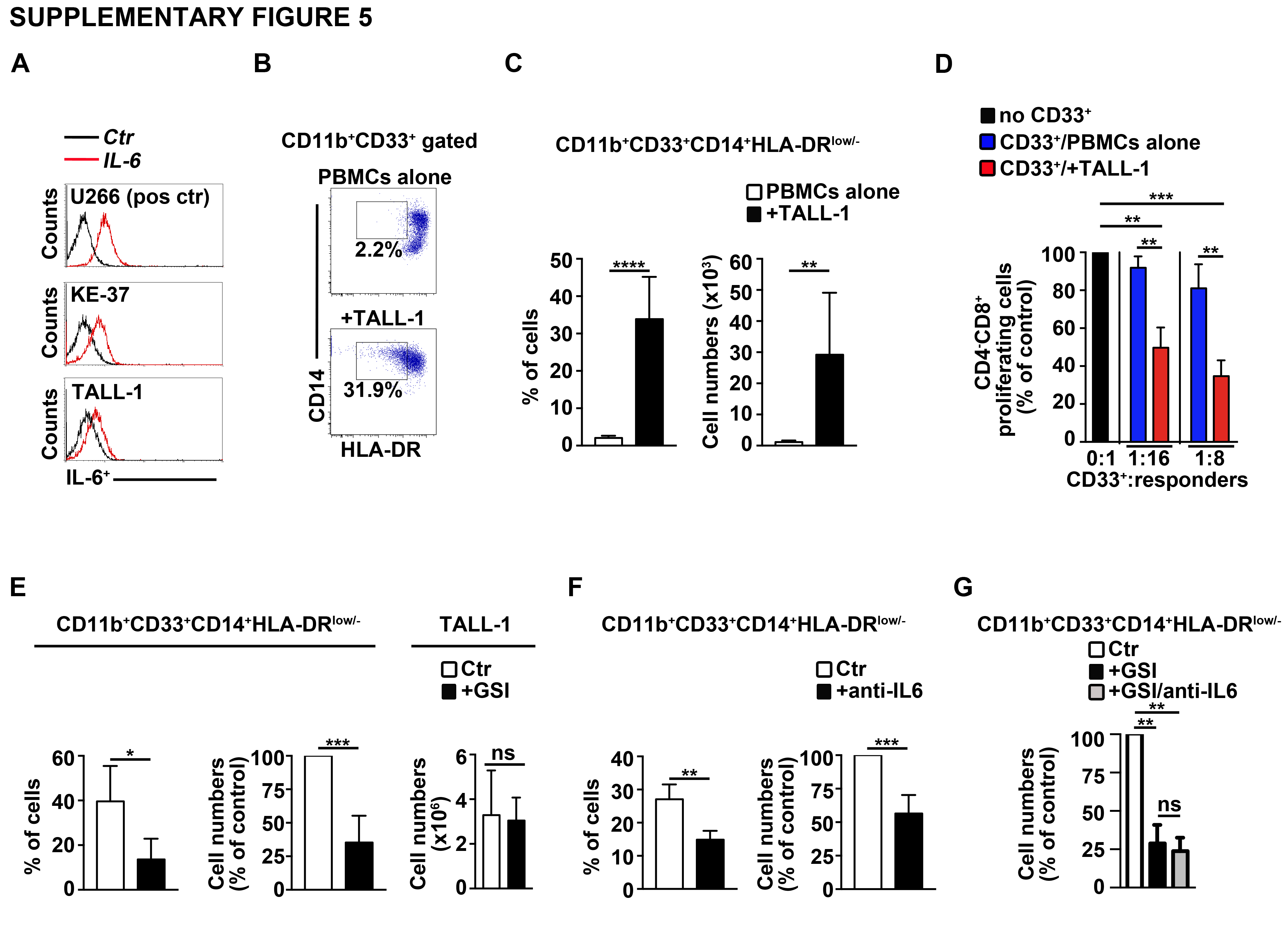
**

**Supplementary Figure 5. The human Notch3-dependent T-ALL cell line, TALL-1 drives the expansion of MDSCs from healthy PBMCs. (A)** FACS analysis of intracellular IL-6 staining in the indicated human T-ALL cell lines (red lines). Black lines=ligand blocking controls (see Materials and methods). The human multiple myeloma U266 cell line was used as a positive control. Data are representative of three independent experiments. PBMCs from healthy donors were co-cultured with the human Notch3-dependent T-ALL cell line, TALL-1, by using transwell inserts, or were cultured in medium alone, as a control, for six days. The data shown are relative to the end of culturing. **(B)** Representative dot plot of CD14^+^HLA-DR^low/neg^ cells inside CD11b^+^CD33^+^ subset, as measured by FACS analysis in harvested PBMCs/TALL-1 co-culture samples (+TALL-1), *versus* samples of autologous PBMCs cultured alone (PBMCs alone). Numbers inside cytograms indicate the percentages of CD11b^+^CD33^+^CD14^+^HLA-DR^low/neg^ cells. **(C)** Percentages (left panel) and absolute numbers (right panel) of CD11b^+^CD33^+^CD14^+^HLA-DR^low/neg^ cells, as measured by the same FACS analysis, as in (B). The values are presented as mean±SD from six independent experiments (n=8 samples per group, donors n=8), each in triplicates. **, P≤0.01 and ****, P ≤ 0.0001 represent significant differences between the indicated groups. **(D)** At the end of the co-culture assay above, the CD33^+^ fractions were magnetically selected from harvested samples and used as 'putative' suppressors (CD33^+^) in a suppression test on proliferating CD4^-^CD8^+^ gated cells ('responders'), from CFSE-labelled and activated autologous PBMCs, at the indicated CD33^+^:responders ratios (0:1, 1:16, 1:8). CD33^+^ cells were selected from PBMCs cultured either in medium alone (CD33^+^/PBMCs alone, blue bars) or with TALL-1 cells (CD33^+^/+TALL-1, red bars). The results are illustrated as the ratio between the percentages of proliferating CD4^-^CD8^+^ gated cells in cultures containing CD33^+^ and in control cultures set up in the absence of CD33^+^ (no CD33^+^, black bar), expressed as % of control, and assessed as CFSE dilution by FACS analysis at 72 h of culturing. The values are presented as the mean±SD from three independent experiments (n=3 samples per group, donors n=3), with three technical replicates per experiment. **, P≤0.01 and ***, P≤ 0.001 represent significant differences between the indicated groups. **(E)** Percentages (left panel) and cell count proportions (middle panel) of CD11b^+^CD33^+^CD14^+^HLA-DR^low/neg^ cells in harvested PBMCs/TALL-1 co-culture samples, in the absence (CTR, white bars) or presence of GSI (+GSI, black bars), as measured by the same FACS analysis, as in (B). The right panel shows the absolute numbers of TALL-1 cells from the same co-cultures, as counted at the end of the co-culturing time. **(F)** Percentages (left panel) and cell count proportions (right panel) of CD11b^+^CD33^+^CD14^+^HLA-DR^low/neg^ cells in harvested PBMCs/TALL-1 co-culture samples, in the absence (CTR, white bars) or presence of anti-IL-6 neutralizing antibodies (+anti-IL6, black bars), as measured by the same FACS analysis, as in (B). **(G)** Cell count proportions of CD11b^+^CD33^+^CD14^+^HLA-DR^low/neg^ cells in harvested PBMCs/TALL-1 co-culture samples, in the absence (CTR, white bar) or presence of GSI (+GSI, black bar) or presence of both GSI and anti-IL6 antibodies (+GSI/anti-IL6, grey bar), as measured by the same FACS analysis, as in (B). In (E-G) the cell count proportions were calculated as the ratio between the CD11b^+^CD33^+^CD14^+^HLA-DR^low/neg^ absolute numbers in PBMCs/TALL-1 treated co-culture samples and in relative untreated controls, and expressed as % of control. The values represent the mean±SD from four independent experiments (n=4 samples per group, donors n=4), each in triplicates. ns=not significant, P>0.05; *, P≤0.05; **, P≤0.01 and ***, P≤0.001 represent significant differences between the indicated groups.

**
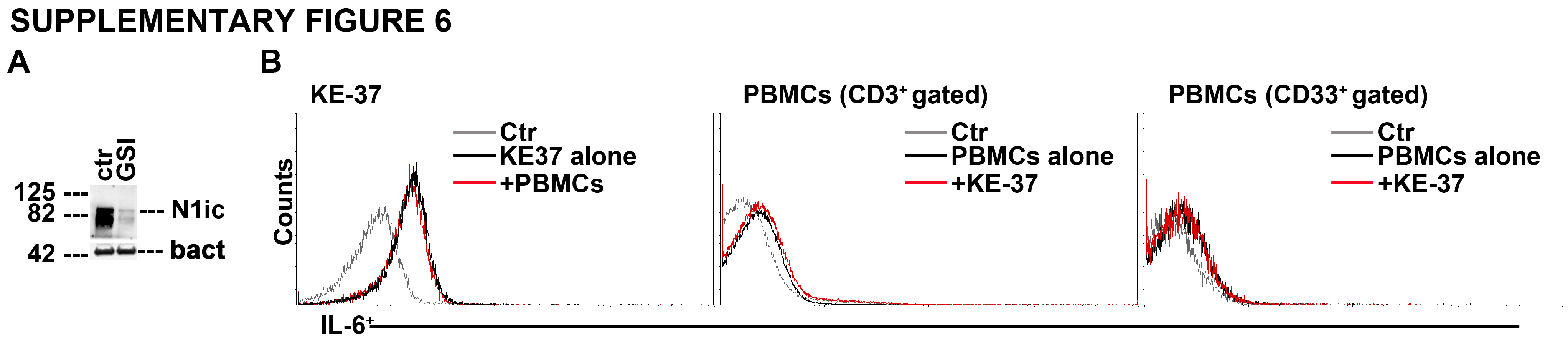
**

**Supplementary Figure 6. During the co-cultures with healthy PBMCs, KE-37 cells express IL-6 and down-modulate Notch1-active protein upon GSI treatment (A)** Western blot analysis of cleaved Notch1 intracellular domain (N1ic) expression in KE-37 cells, at the day six of co-culturing with PBMCs in the presence of GSI 10 μm or DMSO, as a control. Molecular weights in kDa are indicated in the left part. The results are representative of three independent experiments (n=3 samples per group, donors n=3), each in triplicates. (**B**) FACS analysis of IL-6 expression in KE-37 cells (left panel), as well as in CD3^+^ T cell subset (middle panel) and CD33^+^ myeloid cells (right panel) from healthy PBMCs, as measured at day six of culturing alone (black lines) or in PBMCs/KE37 co-cultures (red lines). The grey lines represent staining controls (see methods). Data are representative of three independent experiments (n=3 samples per group, donors n=3), each in duplicates.

**
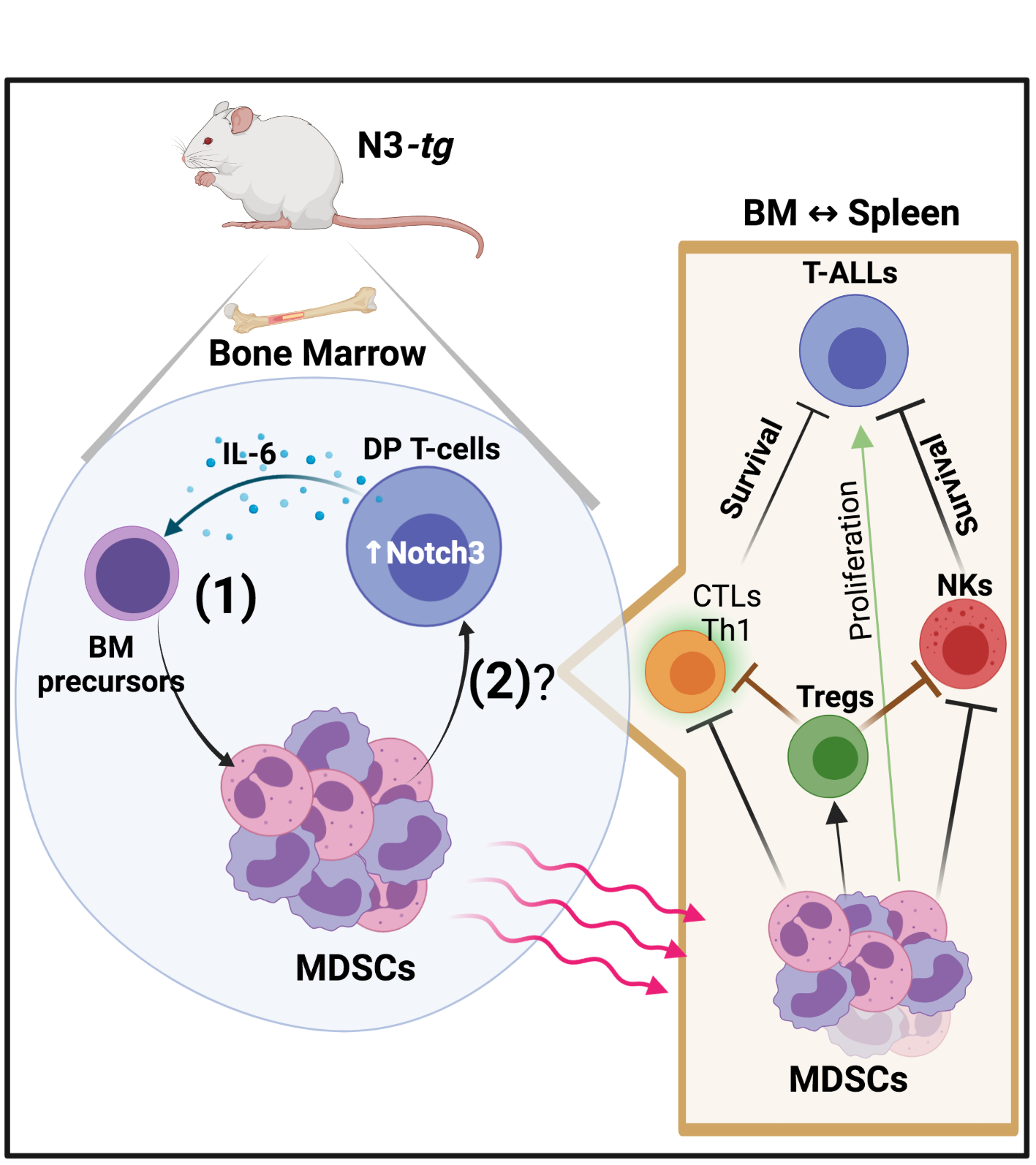
**

**Supplementary Figure 7. The Notch3 dependent-induction of MDSCs and their possible role in T-ALL progression.** On the left part, the figure summarizes the *in vivo* results on our Notch3-dependent model of T-ALL (***N3-tg*** mice). Inside the Bone-Marrow aberrant T cells, mainly represented by CD4^+^CD8^+^ DP T cells (**DP T-cells**) induce functional MDSCs (**MDSCs**) from BM precursors, through a mechanism influenced by Notch-signaling deregulation inside DP T cells **(↑Notch3**) and the presence/release of **IL-6** in the TME **(1).** In turn, MDSCs sustain DP T cell proliferation and/or survival **(2),** and eventually T-ALL progression, through mechanisms that we have hypothesized **(?)** on the right part of the figure (see the rectangle box), and that are likely active in BM of transgenic mice, but also in the spleen (**BM⇔Spleen**), where MDSCs migrate (see the red curve arrows). In particular, MDSC may improve T-ALL cell **(T-ALLs)** survival/proliferation, either directly (green arrow) and, more likely, indirectly by blocking anti-tumor immune responses of **NKs**, besides that of **CTL/Th1** subsets, as well as through the generation of immuno-suppressive **Tregs**. **Created with BioRender.com**
